# Potential cardiotoxicity induced by Euodiae Fructus: *In vivo* and *in vitro* experiments and untargeted metabolomics research

**DOI:** 10.3389/fphar.2022.1028046

**Published:** 2022-10-24

**Authors:** Dan Zhang, Jintao Lü, Zhixin Ren, Xiaomeng Zhang, Huanzhang Wu, Rina Sa, Xiaofang Wang, Yu Wang, Zhijian Lin, Bing Zhang

**Affiliations:** ^1^ School of Chinese Materia Medica, Beijing University of Chinese Medicine, Beijing, China; ^2^ Centre for Pharmacovigilance and Rational Use of Chinese Medicine, Beijing University of Chinese Medicine, Beijing, China; ^3^ Gansu Provincial Hospital, Lanzhou, China

**Keywords:** Euodiae Fructus, cardiotoxicity, H9c2, neonatal rat cardiomyocytes, molecular mechanism, untargeted metabolomics

## Abstract

**Background:** Euodiae Fructus, a well-known herbal medicine, is widely used in Asia and has also gained in popularity in Western countries over the last decades. It has known side effects, which have been observed in clinical settings, but few studies have reported on its cardiotoxicity.

**Methods:** In the present study, experiments using techniques of untargeted metabolomics clarify the hazardous effects of Euodiae Fructus on cardiac function and metabolism in rats in situations of overdosage and unsuitable syndrome differentiation. *In vitro* assays are conducted to observe the toxic effects of evodiamine and rutaecarpine, two main chemical constituents of Euodiae Fructus, in H9c2 and neonatal rat cardiomyocytes (NRCMs), with their signaling mechanisms analyzed accordingly.

**Results:** The cardiac cytotoxicity of evodiamine and rutaecarpine in *in vivo* experiments is associated with remarkable alterations in lactate dehydrogenase, creatine kinase, and mitochondrial membrane potential; also with increased intensity of calcium fluorescence, decreased protein expression of the cGMP-PKG pathway in H9c2 cells, and frequency of spontaneous beat in NRCMs. Additionally, the results in rats with Yin deficiency receiving a high-dosage of Euodiae Fructus suggest obvious cardiac physiological dysfunction, abnormal electrocardiogram, pathological injuries, and decreased expression of PKG protein. At the level of endogenous metabolites, the cardiac side effects of overdose and irrational usage of Euodiae Fructus relate to 34 differential metabolites and 10 metabolic pathways involving among others, the purine metabolism, the glycerophospholipid metabolism, the glycerolipid metabolism, and the sphingolipid metabolism.

**Conclusion:** These findings shed new light on the cardiotoxicity induced by Euodiae Fructus, which might be associated with overdose and unsuitable syndrome differentiation, that comes from modulating the cGMP-PKG pathway and disturbing the metabolic pathways of purine, lipid, and amino acid. Continuing research is needed to ensure pharmacovigilance for the safe administration of Chinese herbs in the future.

## 1 Introduction

Euodiae Fructus, commonly known as “Wuzhuyu” in Chinese, is a potent internal-warming traditional herbal medicine, and has been extensively used in clinical treatment due to its analgesic, antiemetic, anti-inflammatory, antidiarrheal, neuroprotective, and cardioprotective activities (Lee et al., 2011; Liao et al., 2011; Cai et al., 2014; Li and Wang, 2020). Although Euodiae Fructus has demonstrated promising therapeutic effects for headaches, abdominal pain, diarrhea, and vomiting induced by pathogenic cold, its potential cardiotoxicity has also been recently recognized (Zeng and Jiang, 2010; Yang et al., 2017). With regard to the herb itself, potential cardiotoxicity might be related to bioactive substances with the dual characteristics of efficacy and toxicity, such as evodiamine and rutaecarpine. On the one hand, evodiamine and rutaecarpine can produce beneficial pharmacodynamic and pharmacological effects for anti-arrhythmia, myocardial protection and recovery, as evidenced by previous research based on experiments around isolated atria in guinea pigs, cardiac fibrosis in mice, and myocardial ischemia-reperfusion injury and cardiac hypertrophy in rats (Kobayashi et al., 2001; Rang et al., 2004; Jiang et al., 2017; Tian et al., 2019; Li et al., 2021; Zhan et al., 2021). On the other hand, the toxicological effects of evodiamine on the heart, which might be associated with oxidative stress, have been observed through *in vivo* and *in vitro* experiments with primary neonatal rat cardiomyocytes and zebra fish (Yang et al., 2017). In addition, dehydroevodiamine and hortiamine might be responsible for potential proarrhythmic effects, because they have been identified from the extract of Euodiae Fructus as hERG inhibitors *via* the technologies of HPLC-microfractionation, patch clamp, and so on (Zhan et al., 2021).

It is worth noting that irrational use of TCM herbs, including overdose, self-medication, and so forth, can occasionally induce serious adverse reactions or even fatal poisoning (Zhang et al., 2012; Chan et al., 2015; Li et al., 2018a; Zhang et al., 2020). The distinct cardiovascular activity of Euodiae Fructus might thus be transformed into underlying cardiac toxicity under different physiological, pathological, and clinical conditions, with overdose and unsuitable syndrome differentiation contributing to the cardiac risk. Despite the large number of studies focusing on the herb-related adverse reactions and corresponding mechanisms of Euodiae Fructus, the current profiles of the cardiac toxicity of Euodiae Fructus are not well delineated (Cai et al., 2014; Zhang et al., 2015; Pan et al., 2020). There is overwhelming research evidence that the cGMP-PKG pathway in the heart plays a principal role in regulating myocardial function and electrophysiology through multiple downstream targets, involving the G-protein coupled receptor, the calcium signaling pathway, and so on (Inserte and Garcia-Dorado, 2015; Park et al., 2018; Nakamura and Tsujita, 2021). Given this, advanced and comprehensive methodologies were applied in *in vivo* and *in vitro* experiments and in untargeted metabolomics, such as electrocardiograms (ECGs), serum biomarkers, histopathology, and metabolomics, to better characterize the manifestations of cardiac toxicity in H9c2 cells, neonatal rat cardiomyocytes (NRCMs), and rats, and to further illustrate the signaling mechanisms and endogenous metabolites for the related poisoning.

## 2 Materials and methods

**FIGURE 1:**
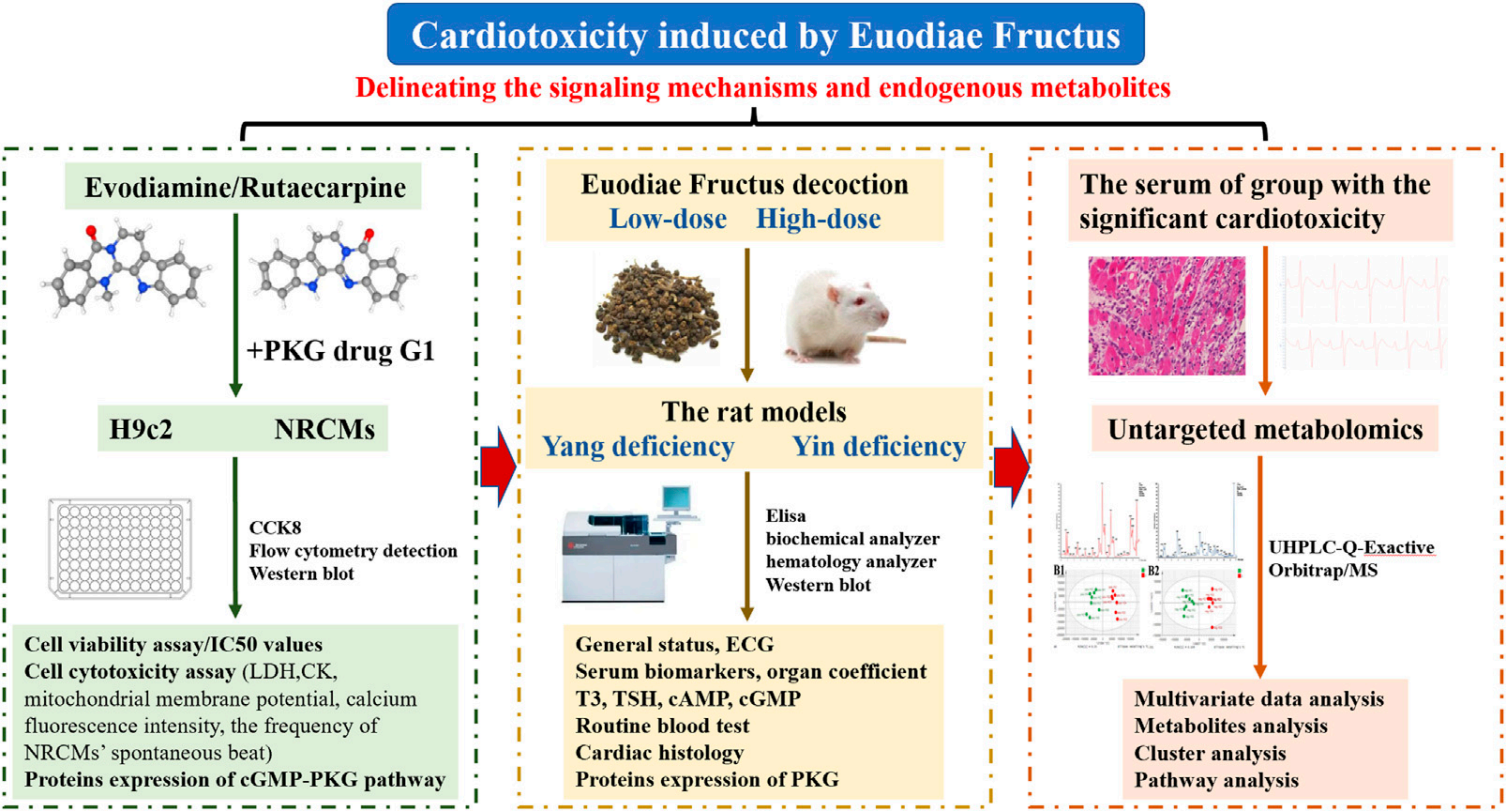
The flowchart of the technical strategy in the present study.

The present study, focusing on the cardiotoxicity induced by Euodiae Fructus, was conducted by cell experiments *in vitro* of H9c2 and NRCMs, by experiments *in vivo* of the model of rats with either Yang or Yin deficiency, and by untargeted metabolomics research on the serum of the group with significant cardiotoxicity (Figure 1).

### 2.1 *In vitro* experiments: Cardiotoxicity from evodiamine and rutaecarpine in H9c2 cells

#### 2.1.1 Cell culture

Rat cardiomyocyte-derived H9c2 cells from the National Infrastructure of Cell Line Resources (Chinese Academy of Medical Sciences, Beijing, China) were cultivated in Dulbecco’s Modified Eagle Medium (DMEM), high glucose (Biological Industries, Israel) supplemented with 10% fetal bovine serum (FBS, Biological Industries, Israel), 1% penicillin-streptomycin (Corning, United States) at 37°C in a 5% CO_2_ atmosphere. *In vitro* experiments were performed using H9c2 cardiomyocytes between passages 15 and 20, which were subcultured at a confluence of approximately 80%.

#### 2.1.2 Cell viability assay

H9c2 cardiomyocytes (5,000/well) were cultured into 96-well plates for 24 h, and were exposed to a series concentration of evodiamine and rutaecarpine (Shanghai Yuanye Bio-Technology Co., Ltd., China) for another 24 h. Cell viability was measured using the cell counting kit-8 (CCK-8) solution assay (Biorigin Inc., China) at 450 nm. Subsequently, the absorbance values were applied to calculate the half maximal inhibitory concentration (IC_50_ values) and select appropriate concentrations for further experiments. All experiments were performed independently in triplicate.

#### 2.1.3 Cell cytotoxicity assay

After incubation with different concentrations of evodiamine (5, 10, 25 μM) and rutaecarpine (60, 80, 100 μM) for 24 h, according to the manufacturer’s directions, the leakage of lactate dehydrogenase (LDH) and the activity of creatine kinase (CK) were determined using the commercial LDH and CK detection kits (Nanjing Jiancheng Bioengineering Institute, China), respectively. Additionally, the mitochondrial membrane potential and the intensity of calcium fluorescence were evaluated with the JC-1 and Fluo-3AM detection kit (Beyotime Biotechnology, China) through FACS Calibur flow cytometry detection (Becton, Dickinson and Company, United States).

#### 2.1.4 Western blot analysis

The proteins of H9c2 cardiomyocytes from different groups were harvested and lysed with cold RIPA buffer (Beijing Solarbio Technology Co., Ltd., China), supplemented with a protease inhibitor cocktail for 15 min on ice, and the concentrations of the supernatant were measured with a BCA protein assay kit (Beijing Solarbio Technology Co., Ltd., China). Briefly, equal amounts (10 µg) of protein were separated *via* pre-cast 8% SDS-polyacrylamide gel and transferred onto polyvinylidene difluoride (PVDF) membranes (Millipore Inc., United States). After blocking with TBST containing 5% skim milk for 1 h at room temperature, the PVDF membranes were incubated overnight at 4°C with PRKG1 antibody (1:1,000, Proteintech Group, Inc., United States), cGMP antibody (1:1,000, Santa Cruz Biotechnology, United States), and GAPDH antibody (1:30,000, Proteintech Group, Inc., United States), followed by incubation with the appropriate secondary antibodies at room temperature for another 1 h. Ultimately, all the blots were visualized by SageCapture software (Beijing Sage Creation Science Compony, China), the levels of protein expression were normalized to that of GAPDH, and relative protein expression was quantified by utilizing Image-ProPlus 6.0 software (Media Cybernetics, United States). Western blots were performed at least three times.

### 2.2 *In vitro* experiments: Cardiotoxicity from evodiamine and rutaecarpine plus the PKG drug G1 in H9c2 cells

The PKG drug G1 (Selleck Chemicals LLC, United States), the activator of protein kinase G Iα (PKG Iα) was used as tool to further research the function of PKG protein for cardiac toxicity induced by evodiamine and rutaecarpine in H9c2 cells (Burgoyne et al., 2017; Maset et al., 2021). Based on the cell viability of H9c2 cardiomyocytes and the expression of PKG, the optimal concentration of the activator was detected for follow-up studies.

In the aforementioned process, the cell cytotoxicity assay was conducted to include LDH leakage, CK activity, mitochondrial membrane potential, and the intensity of calcium fluorescence, while related protein expression was measured for H9c2 cells exposed to evodiamine and rutaecarpine plus the PKG drug G1.

### 2.3 *In vitro* experiments: Cardiotoxicity from evodiamine and rutaecarpine in NRCMs

#### 2.3.1 Cell culture

Given the limitations of H9c2 cardiomyocytes, neonatal rat cardiomyocytes (NRCMs), considered common models for studying the morphological, biochemical, and electrophysiological characteristics of the heart (Chlopcikova et al., 2001), were obtained from 2-3 day-old Sprague–Dawley (SD) rats (Beijing Si Pei Fu Biotechnology, Certificate SCXK-2019-0010) after strict sterilization by the methodology used for isolation and cultivation in previous research, with some modifications (Sabri et al., 2003; Rafiq et al., 2006; Shukla et al., 2018). The apex of isolated heart tissue was digested repeatedly in the short term in a mixture of collagenase II (Biorigin Inc., China) and 0.25% trypsin (Gibco Life Technologies, China) with a magnetic stirrer at 37°C. The cells were incubated in DMEM, supplemented with 15% FBS and 1% penicillin-streptomycin for 1 h. There were fibroblasts adhering to the wall, and the supernatant was resuspended in 96-well plates at a density of 5 × 10^5^ cells/ml, while 100 μM 5-bromo-2-deoxyuridine (BrdU, Biorigin Inc., China) was added to the culture medium to inhibit fibroblast proliferation. These non-adherent cells were incubated at 37°C under humidified conditions of 5% CO_2_ for 24 h, and the medium was replaced. On days 4 to 5 of culture, confluent monolayers of NRCMs with regular spontaneous contractility were used for the observation of cardiac toxicity induced by evodiamine and rutaecarpine (Frolova et al., 2016; Frolova et al., 2019).

#### 2.3.2 Cell cytotoxicity assay

To detect the influence of spontaneous contractility, the spontaneous beat frequency of the NRCMs was recorded after interventions with evodiamine (5, 10, 25 μM) and rutaecarpine (60, 80, 100 μM) for 15 min, 30 min, 1 h, 2 h, and 4 h, separately. Moreover, the cell viability and the LDH leakage of the NRCMs were detected using corresponding kits after 4 h of administration.

### 2.4 *In vivo* experiments: Cardiotoxicity from Euodiae Fructus in rats

#### 2.4.1 Preparation of reagents and Euodiae Fructus decoction

A hydrocortisone sodium succinate for injection (Tianjin Biochem Pharmaceutical Co., Ltd., China) was diluted with saline to a 20 mg/ml solution for use. The preparation of the 1.5 mg/ml thyroid suspension was made by dissolving oral thyroid tablets (Shanghai Zhonghua Pharmaceutical Co., Ltd., China) in carboxymethylcellulose sodium (CMC-Na, BioRuler Company, United States). In addition, the herbal materials called Euodiae Fructus Praeparata were purchased from Beijing Sanhe Pharmaceutical Co. Ltd (Beijing, China, Lot 12410101), and authenticated by Prof. Chunsheng Liu, Beijing University of Chinese Medicine, as the fruit of *Tetradium ruticarpum* (A. Juss.) T. G. Hartley. The decoction of Euodiae Fructus was boiled twice; 1 kg decoction pieces were decocted with water (1:10 volume) for 45 min the first time, before eight times the amount of water was added for another 30 min. Finally, the supernatants were combined and concentrated into a 0.525 g/ml decoction of Euodiae Fructus.

#### 2.4.2 Experimental design

Adult male SD rats weighing 180 ± 10 g (Beijing Si Pei Fu Biotechnology, Certificate SCXK-2020-0033) were acclimatized for 3 days in the animal facility at Beijing University of Chinese Medicine. The rat models of Yang deficiency and those of Yin deficiency were gavage administered the with the decoction of Euodiae Fructus, whose potential cardiotoxicity was investigated to delineate the signaling mechanisms *in vivo*. All the animal experiments were conducted in accordance with approved guidelines specified by the animal ethics committee of Beijing University of Traditional Chinese Medicine (Beijing, China; No. BUCM-4-2021090302-3052).

The manufacture of rat models with Yang deficiency was achieved by an intragluteal injection of 20 mg/ml hydrocortisone sodium succinate (1 ml/kg), continued for 15 days, as in the previous work of our team (Zhang, 2013). Meanwhile, the rat models with Yin deficiency received gavage administration of 1.5 mg/ml thyroid suspension (10 ml/kg) for 15 days (Zhang et al., 2019). Simultaneously, the medication group received intragastric administration of the decoction of Euodiae Fructus (the low dose was 0.0583 g/ml, the high dose was 0.525 g/ml), based on the modeling of Yang and Yin deficiencies.

All rats were randomly divided into eight groups (*n* = 8/group): 1) the Yang-K group (treated with intragluteal injection of an equal volume of the sterilized saline); 2) the Yang-X group (received intragluteal injection of hydrocortisone sodium succinate 1 ml/kg); 3) the Yang-D group (administered intragluteal injection of hydrocortisone sodium succinate 1 ml/kg + the decoction of Euodiae Fructus 0.0583 g/ml); 4) the Yang-G group (administered intragluteal injection of hydrocortisone sodium succinate 1 ml/kg + the decoction of Euodiae Fructus 0.525 g/ml); 5) the Yin-K group (received gavage administration of water); 6) the Yin-X group (given gavage administration of thyroid suspension 10 ml/kg); 7) the Yin-D group (received gavage administration of thyroid suspension 10 ml/kg + the decoction of Euodiae Fructus 0.0583 g/ml); and 8) the Yang-G group (received gavage administration of thyroid suspension 10 ml/kg + the decoction of Euodiae Fructus 0.525 g/ml).

#### 2.4.3 Observation of general status

The changes in the general status of different groups were observed immediately; the body weights and rectal temperatures of rats were measured 7 days and 14 days after treatment.

#### 2.4.4 Measurement of ECG, serum biomarkers, and organ coefficients

All rats per group were sacrificed on day 15 by anesthetization with an intraperitoneal injection of 10% chloral hydrate (3 ml/kg). After anesthesia, the rats were fixed in a supine position, and the ECG was recorded through a BL-420S biological function experiment system (Chengdu Taimeng Software Co., Ltd., China) to inspect the cardiac function.

Blood was collected from the abdominal aorta for different detection indexes, for which the plasma, serum and whole blood were prepared separately. To explore the cardiac injury, serum biomarkers, including lactate dehydrogenase (LDH), creatine kinase (CK), α-hydroxybutyrate dehydrogenase (HBDH), and aspartate aminotransferase (AST), along with the glucose and lipid metabolism involving glucose (GLU), triacylglycerol (TG), and cholesterol (CHO), were detected using the AU5800 automatic biochemical analyzer (Beckman Coulter, Inc., United States). With regard to the organ coefficients, the organs (including liver, kidneys, heart, spleen, and lungs) of each rat were dissected and weighed, and the hearts were removed for subsequent experimentation.

#### 2.4.5 Measurement of T3 and TSH content in serum, cAMP and cGMP in plasma, and routine blood tests

The content of triiodothyronine (T3) and the thyroid stimulating hormone (TSH) in serum, and cyclic adenosine monophosphate (cAMP) and cyclic guanosine monophosphate (cGMP) in plasma, were determined using related enzyme-linked immunosorbent assay (ELISA) kits (Wuhan Cloud-Clone Corp., China) in accordance with the manufacturer’s instructions. Using the hematology analyzer (Sysmex Corporation, Japan), the routine blood test was conducted on blood samples collected from rats in the different groups, measuring especially white blood cells (WBC), red blood cells (RBC), hemoglobin (HGB), platelets (PLT), neutrophil ratio (NEUT%), lymphocyte ratio (LYMPH%), and monocyte ratio (MONO%).

#### 2.4.6 Cardiac histology

Normal saline was used for irrigating the hearts. Afterward, the hearts were fixed in 10% formalin for 24 h, embedded in paraffin, and sectioned transversely at 4 µm. The histopathological changes of myocardia for rats in different groups were investigated by haematoxylin-eosin (HE) staining.

#### 2.4.7 Western blot analysis

Total proteins from the heart tissue of rats were homogenized and extracted, and the expression of PKG protein was examined through western blot analysis, according to related procedures of *in vitro* experimentation.

### 2.5 Untargeted metabolomics: Cardiotoxicity from Euodiae Fructus in rats

#### 2.5.1 Sample preparation

For the group with significant cardiotoxicity, the endogenous metabolites in the serum were investigated using the approach of ultra-high performance liquid chromatography quadrupole-exactive Orbitrap/mass spectrum (UHPLC-Q-Exactive Orbitrap/MS), as described previously (Liu et al., 2019).

Briefly, aliquots (100 μl) of plasma samples were mixed with 300 μl chromatographic acetonitrile. After centrifugation (13,000 rpm, 15 min, 4°C), the supernatant was transferred to a clean tube for analysis. For methodological investigations, the quality control (QC) samples were prepared from mixtures of 10 μl plasma in each sample.

#### 2.5.2 Sample detection

Aliquots (2 μl) of experimental samples were eluted through an ACQUITY UPLC BEH C18 chromatographic column (2.1 mm × 100 mm, 1.7 µm, Waters Corporation, United States) in a Vanquish Duo UHPLC chromatograph (Thermo Fisher Scientific Inc., United States), using the mobile phases of eluents A (acetonitrile) and B (0.1% formic acid in water) at a flow rate of 0.3 ml/min.

Electron spray ionization was employed for detecting both positive and negative ions in the abovementioned plasma samples *via* a hybrid quadrupole Orbitrap mass spectrometer (Q Exactive, Thermo Fisher Scientific Inc., United States). The quadrupole scan range was set at mass-to-charge ratio (m/z) 100–1,200 Da, with the heated capillary temperature at 350°C, and the positive and negative spray voltages at 3.2 and 3.8 kV, respectively.

#### 2.5.3 Multivariate data analysis

The raw data from the liquid chromatography-mass spectrometry (LC-MS) were manually phase-baseline corrected for peak area (PA) and retention time (RT) using the Mass Spectrometry-Data Independent Analysis software version 4 (MS-DIAL 4, http://prime.psc.riken.jp/compms/msdial/main.html) (Tsugawa et al., 2019; Tsugawa et al., 2020). Thereafter the multivariate data analysis was performed with SIMCA-P software (Version 14.1, Umetrics, Umea, Sweden), including principal component analysis (PCA) and the orthogonal partial least square-discriminate (OPLS-DA). Here PCA was a non-supervised approach to observe the distribution and outliers of the data set depicted in a scores plot based on orthogonal latent variables, which were obtained from the overall direction of maximum variance (Duan et al., 2018). Furthermore, owing to supervised algorithms, OPLS-DA was employed to extract the underlying variability in behavior characterizing the endogenous metabolomics. The evaluation methods of the OPLS-DA model were described by the Q^2^ and *R* ^2^ of the permutation respectively. The robustness of the model’s prediction ability is directly proportional to the Q^2^ (0 < Q^2^ < 1), while the *R* ^2^ could represent the percentage of X and Y matrix information of the model interpretation (Triba et al., 2015; Li et al., 2018b; Jang et al., 2018; Plazas et al., 2019).

#### 2.5.4 Metabolites analysis

The most discriminant variables were selected in terms of variable importance in the projection (VIP) with significant statistical difference in the corresponding PA. On the one hand, discriminant metabolites (VIP >1.0) were collected according to related results of OPLS-DA. On the other, the statistical tests were exhibited by SPSS software. The normality of data, considered as an adjusted *p*-value > 0.05, was determined by a Kolmogorov–Smirnov test for each group. With regard to normal and homoscedastic variables, statistical significance was determined using a one-way ANOVA. Otherwise, the differences between groups were determined using the Kruskal–Wallis test, and the significance was considered as a *p*-value below 0.05.

Subsequently, corresponding metabolites were identified according to the Human Metabolome Database (HMDB, http://www.hmdb.ca/) (Wishart et al., 2018). As directly displayed in heatmaps for the content and correlation of identified metabolites, the cluster analysis was constructed using MetaboAnalyst 3.0 (http://www.metaboanalyst.ca/) (Chong et al., 2019), and the results of the pathway analysis for the differential metabolites in rats were visualized in a bubble chart, with the size of the bubble proportional to the importance of the pathway (Chong et al., 2018; Chong and Xia, 2020).

## 3 Results

### 3.1 Cardiotoxicity induced by evodiamine and rutaecarpine in H9c2 cells

#### 3.1.1 Cell viability assay and IC_50_ of evodiamine and rutaecarpine

Compared with the control group, both evodiamine and rutaecarpine presented inhibitory effects in a dose-dependent manner for the cell viability of H9c2 cardiomyocytes. The IC_50_ values of evodiamine and rutaecarpine separately were 42.82 ± 7.55 and 117.97 ± 9.69 μmol/L, and the related details are summarized in Table 1.

**TABLE 1:** Cell viability assay and IC_50_ of evodiamine and rutaecarpine (*n* = 6, ‾*x* ± *s*).

Groups	Concentration (μmol/L)	Cell viability (%)	IC_50_ (μmol/L)
Evodiamine	0	100.00 ± 0.014	42.82 ± 7.55
	2	74.43 ± 2.79	
	5	68.50 ± 4.25	
	10	71.03 ± 2.65	
	25	63.95 ± 8.60*	
	50	51.64 ± 12.39**	
Rutaecarpine	0	100.00 ± 7.87	117.97 ± 9.69
	20	112.57 ± 8.22	
	40	103.16 ± 10.75	
	60	106.68 ± 15.89	
	80	88.05 ± 15.86	
	100	63.17 ± 7.59	

Note: Compared with the control group, *p < 0.05; **p < 0.01.

#### 3.1.2 Cell cytotoxicity assay of evodiamine and rutaecarpine in H9c2

**FIGURE 2:**
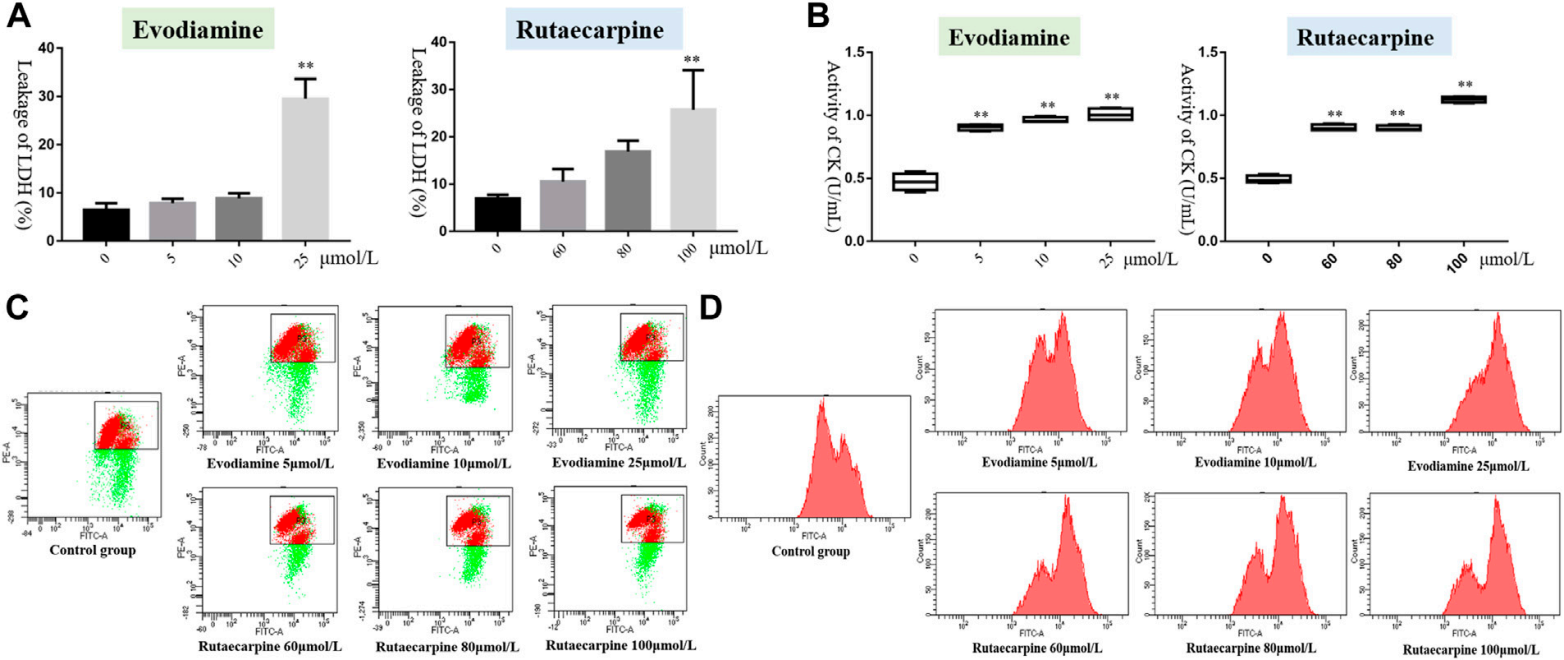
Cell cytotoxicity assay of evodiamine and rutaecarpine in H9c2. Note: **(A)** leakage of LDH; **(B)** activity of CK (U/ml); **(C)** mitochondrial membrane potential; **(D)** intensity of calcium fluorescence.

According to the results of Figure 2; Table 2, the leakage of LDH and the activity of CK were notably more highly dose-dependent in the high-dose evodiamine and rutaecarpine group than in the control group (*p* < 0.01). Similarly, the intensity of calcium fluorescence for H9c2 cells in the high-dose evodiamine and rutaecarpine group was obviously higher (*p* < 0.05). However, significant differences were only observed in the evodiamine-induced H9c2 cells compared to the control group (*p* < 0.01). These results indicate that evodiamine and rutaecarpine might change the permeability of the myocardial cell, the activity of the myocardial enzyme, the energy supply, and the calcium concentration, thereby inducing cardiotoxicity of H9c2 cardiomyocytes.

**TABLE 2:** Cell cytotoxicity assay of evodiamine and rutaecarpine in H9c2 (*n* = 4, ‾*x* ± *s*).

Groups	Concentration (μmol/L)	Leakage of LDH (%) (*n* = 6)	Activity of CK (U/ml)	Mitochondrial membrane potential	Intensity of calcium fluorescence
Evodiamine	0	6.40 ± 1.21	0.47 ± 0.058	1.42 ± 0.025	9111.00 ± 693.42
	5	7.90 ± 0.73	0.90 ± 0.019**	1.44 ± 0.0090	9898.67 ± 677.27
	10	8.89 ± 0.90	0.96 ± 0.019**	1.26 ± 0.0088**	10612.33 ± 746.80
	25	29.54 ± 3.56**	1.01 ± 0.044**	1.11 ± 0.051**	12735.00 ± 594.64**
Rutaecarpine	0	6.91 ± 0.71	0.49 ± 0.025	1.42 ± 0.025	9111.00 ± 693.42
	60	10.54 ± 2.28	0.90 ± 0.022**	1.40 ± 0.028	10731.67 ± 666.35
	80	16.93 ± 1.99	0.90 ± 0.018**	1.39 ± 0.030	11113.00 ± 532.09*
	100	25.74 ± 7.23**	1.13 ± 0.021**	1.31 ± 0.074	12713.67 ± 339.22**

Note: Compared with the control group, *p < 0.05, **p < 0.01.

#### 3.1.3 Protein expression of the cGMP-PKG pathway of evodiamine and rutaecarpine

**FIGURE 3:**
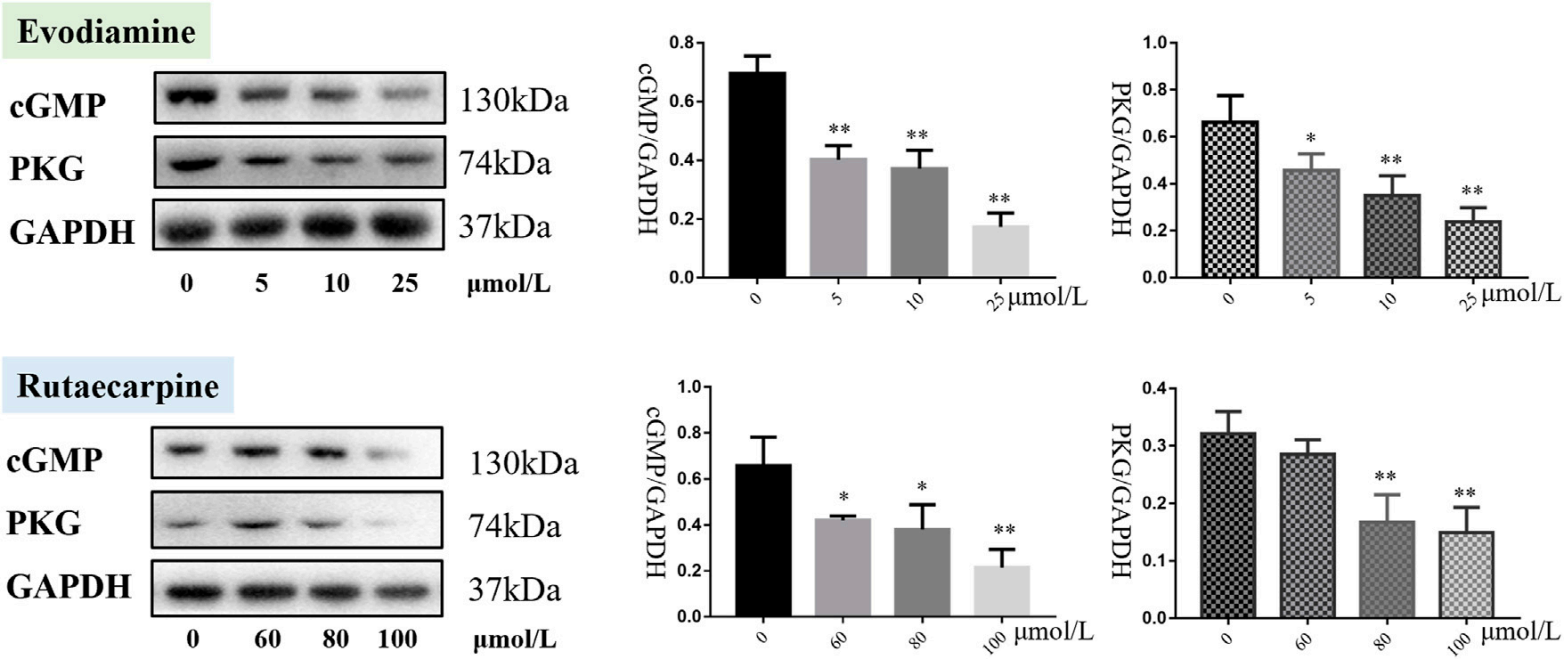
Protein expression of cGMP-PKG pathway of evodiamine and rutaecarpine.

As presented in Figure 3, cGMP and PKG were downregulated in the H9c2 cardiomyocytes with evodiamine (5–25 μmol/L) and rutaecarpine (80–100 μmol/L), compared with the control group (*p* < 0.05), suggesting that the gene and protein expression levels of cGMP and PKG were significantly decreased in H9c2 cardiomyocytes under evodiamine and rutaecarpine (Supplementary Material).

### 3.2 Cardiotoxicity induced by evodiamine and rutaecarpine plus PKG drug G1 in H9c2 cells

#### 3.2.1 Cell viability and cytotoxicity assay of evodiamine and rutaecarpine plus PKG drug G1

**FIGURE 4:**
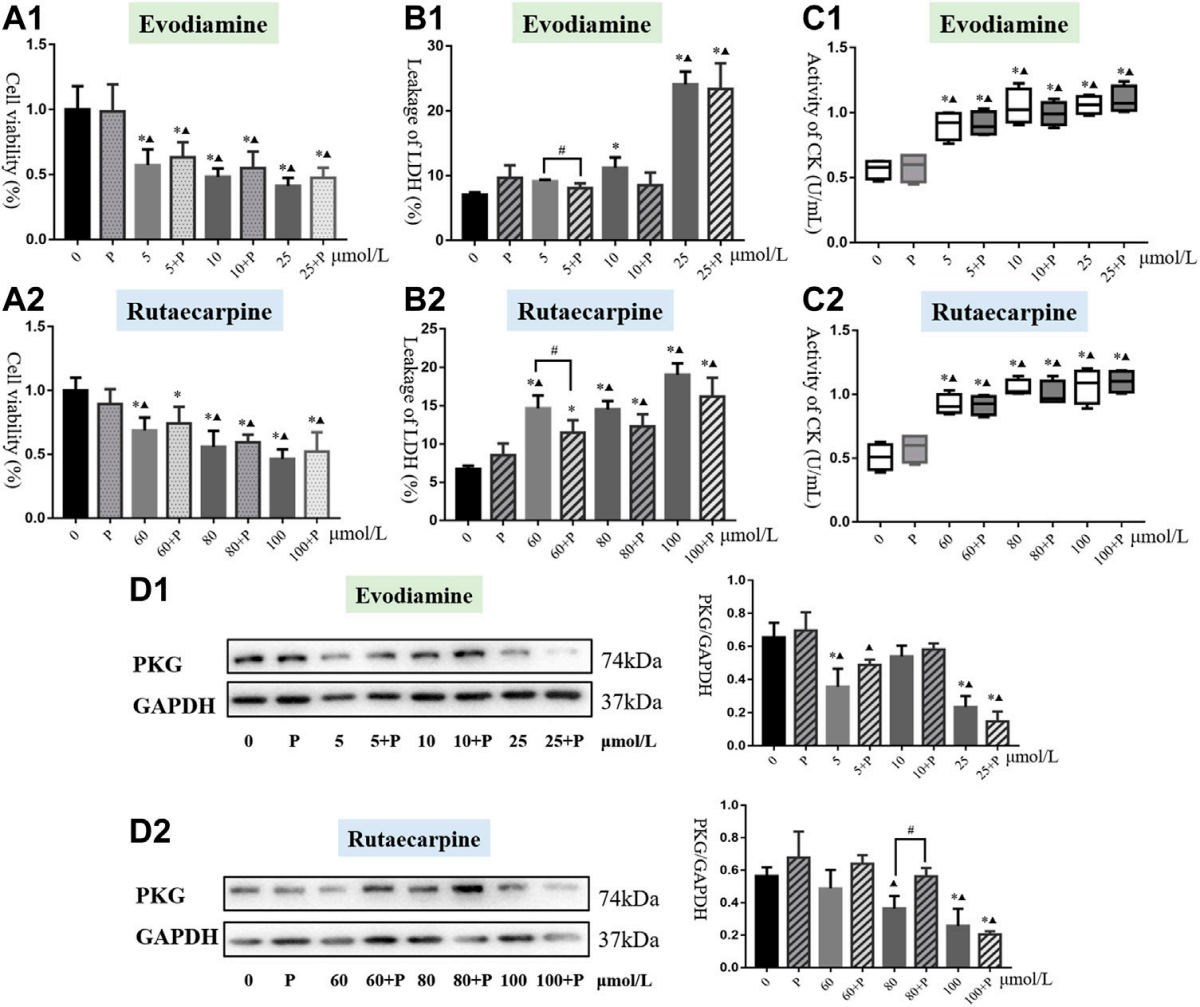
Results of cardiotoxicity induced by evodiamine and rutaecarpine plus PKG drug G1 in H9c2 cells. Note: **(A)** cell viability; **(B)** leakage of LDH; **(C)** activity of CK; **(D)** the protein expression of cGMP and PKG.

The cell viability of each group was apparently lower than in the non-medication group (*p* < 0.05). Additionally, compared with the PKG drug G1 group, only the 60 μmol/L rutaecarpine group was without significant inhibition of H9c2 cardiomyocytes, which means the combination of the PKG drug G1 with evodiamine or rutaecarpine could not have had an appreciable effect on the cell viability of H9c2 cardiomyocytes (Figure 4, Supplementary Material).

As shown in Figure 4, the PKG drug G1 could significantly reduce the leakage of LDH in the low-dose evodiamine and rutaecarpine groups of H9c2 cardiomyocytes, compared with the single agent group (*p* < 0.05). Meanwhile, treatment of the PKG drug G1 obviously improved the mitochondrial membrane potential in the group of 80 μmol/L rutaecarpine (*p* < 0.05), and there were no significant differences for the activity of CK and the intensity of calcium fluorescence between the combined group and the single agent group (Table 3). These results indicate that the PKG drug G1 might partially decelerate the cardiotoxicity of H9c2 cardiomyocytes caused by evodiamine and rutaecarpine.

**TABLE 3:** Cell cytotoxicity of evodiamine and rutaecarpine plus PKG drug G1 (*n* = 4, ‾*x* ± *s*).

Groups	Concentration (μmol/L)	Activity of CK (U/ml)	Mitochondrial membrane potential	Intensity of calcium fluorescence
Control	—	0.51 ± 0.088	1.48 ± 0.110	8852.33 ± 628.59
PKG drug G1	5	0.58 ± 0.097	1.49 ± 0.140	8334.00 ± 693.74
Evodiamine	5	0.90 ± 0.095*^▲^	1.27 ± 0.110	8674.67 ± 465.49
	5 + P	0.91 ± 0.079*^▲^	1.48 ± 0.110	8413.67 ± 510.99
	10	1.04 ± 0.120*^▲^	1.32 ± 0.150	9112.67 ± 501.60
	10 + P	0.99 ± 0.079*^▲^	1.46 ± 0.280	8915.67 ± 709.40
	25	1.06 ± 0.059*^▲^	1.25 ± 0.095	9875.00 ± 730.78
	25 + P	1.10 ± 0.088*^▲^	1.37 ± 0.079	8892.67 ± 472.79
Rutaecarpine	60	0.92 ± 0.068*^▲^	1.48 ± 0.140	9541.67 ± 678.66
	60 + P	0.92 ± 0.065*^▲^	1.45 ± 0.120	8758.00 ± 462.91
	80	1.05 ± 0.054*^▲^	1.28 ± 0.052	9703.67 ± 330.53
	80 + P	1.00 ± 0.082*^▲^	1.45 ± 0.049^#^	8576.33 ± 668.48
	100	1.15 ± 0.075*^▲^	1.23 ± 0.078	10,322.67 ± 428.60^▲^
	100 + P	1.17 ± 0.090*^▲^	1.31 ± 0.190	10,064.67 ± 377.77^▲^

Note: Compared with the control group (non-medication), *p < 0.05; compared with PKG, drug G1 group, ▲p < 0.05; compared with single compound group (corresponding dose), #p < 0.05: P represents 5 μmol/L PKG, drug G1.

#### 3.2.2 Protein expression of PKG from evodiamine and rutaecarpine plus PKG drug G1

As demonstrated by western blot analysis (Figure 4, Supplementary Material), compared with single compound groups, there was an increasing trend of protein expression of PKG in compatibility groups. Remarkably, the PKG drug G1 could greatly enhance the expression of PKG for H9c2 cardiomyocytes incubating with 80 μmol/L rutaecarpine (*p* < 0.05). The inhibitory effects of rutaecarpine (80 μmol/L) were antagonized in concentration-dependent ways by treatment with the PKG drug G1 at concentrations of 5 mol/L.

### 3.3 Cardiotoxicity induced by evodiamine and rutaecarpine in NRCMs

Through observation of NRCMs, the frequency of spontaneous beat in the evodiamine and rutaecarpine groups underwent obvious changes compared with the control group (*p* < 0.05): notably, high-dosage and long-term intervention were associated with cardiomyocyte arrest (Table 4). A significant elevation of the LDH leakage of NRCMs was determined in the evodiamine and rutaecarpine groups compared with the control group over 4 h, whereas cell viability decreased, as listed in Table 5. Consequently, combined with the above results, evodiamine and rutaecarpine might affect the myocardial contractility and normal physiological state of NRCMs.

**TABLE 4:** Frequency of NRCM spontaneous beat of evodiamine and rutaecarpine (*n* = 3, ‾*x* ± *s*).

Groups	Concentration (μmol/L)	15 min	30 min	1 h	2 h	4 h
Control	0	72.67 ± 1.70	72.33 ± 4.18	68.00 ± 1.63	67.67 ± 1.25	69.00 ± 2.94
Evodiamine	5	105.67 ± 4.19**	94.67 ± 1.25**	95.00 ± 1.63**	93.33 ± 1.70**	87.67 ± 2.05**
	10	95.33 ± 1.25**	93.33 ± 2.49**	93.67 ± 2.87**	85.67 ± 2.62**	77.33 ± 2.62**
	25	94.00 ± 2.94**	92.67 ± 2.05**	94.33 ± 2.49**	81.67 ± 4.11**	—
Rutaecarpine	60	106.33 ± 3.30**	94.33 ± 3.09**	82.33 ± 1.25**	81.67 ± 1.25**	76.67 ± 0.94**
	80	101.00 ± 3.56**	92.33 ± 2.49**	91.00 ± 1.63**	75.67 ± 1.25*	—
	100	100.67 ± 2.62**	83.67 ± 4.19*	85.33 ± 3.86**	56.00 ± 4.08**	—

Note: Compared with the control group, *p < 0.05, **p < 0.01; “/” represents cardiomyocyte arrest.

**TABLE 5:** Cardiotoxicity induced by evodiamine and rutaecarpine in NRCMs (*n* = 6, ‾*x* ± *s*).

Groups	Concentration (μmol/L)	Cell viability (%)	Leakage of LDH (%)
Control	0	1.01 ± 0.056	8.70 ± 1.52
Evodiamine	5	0.61 ± 0.084**	22.67 ± 1.58**
	10	0.57 ± 0.055**	26.61 ± 3.74**
	25	0.58 ± 0.090**	26.16 ± 2.96**
Rutaecarpine	60	0.55 ± 0.092**	33.98 ± 4.81**
	80	0.52 ± 0.074**	35.54 ± 4.46**
	100	0.54 ± 0.054**	34.67 ± 2.96**

### 3.4 Cardiotoxicity induced by Euodiae Fructus in rats

#### 3.4.1 General status

**FIGURE 5:**
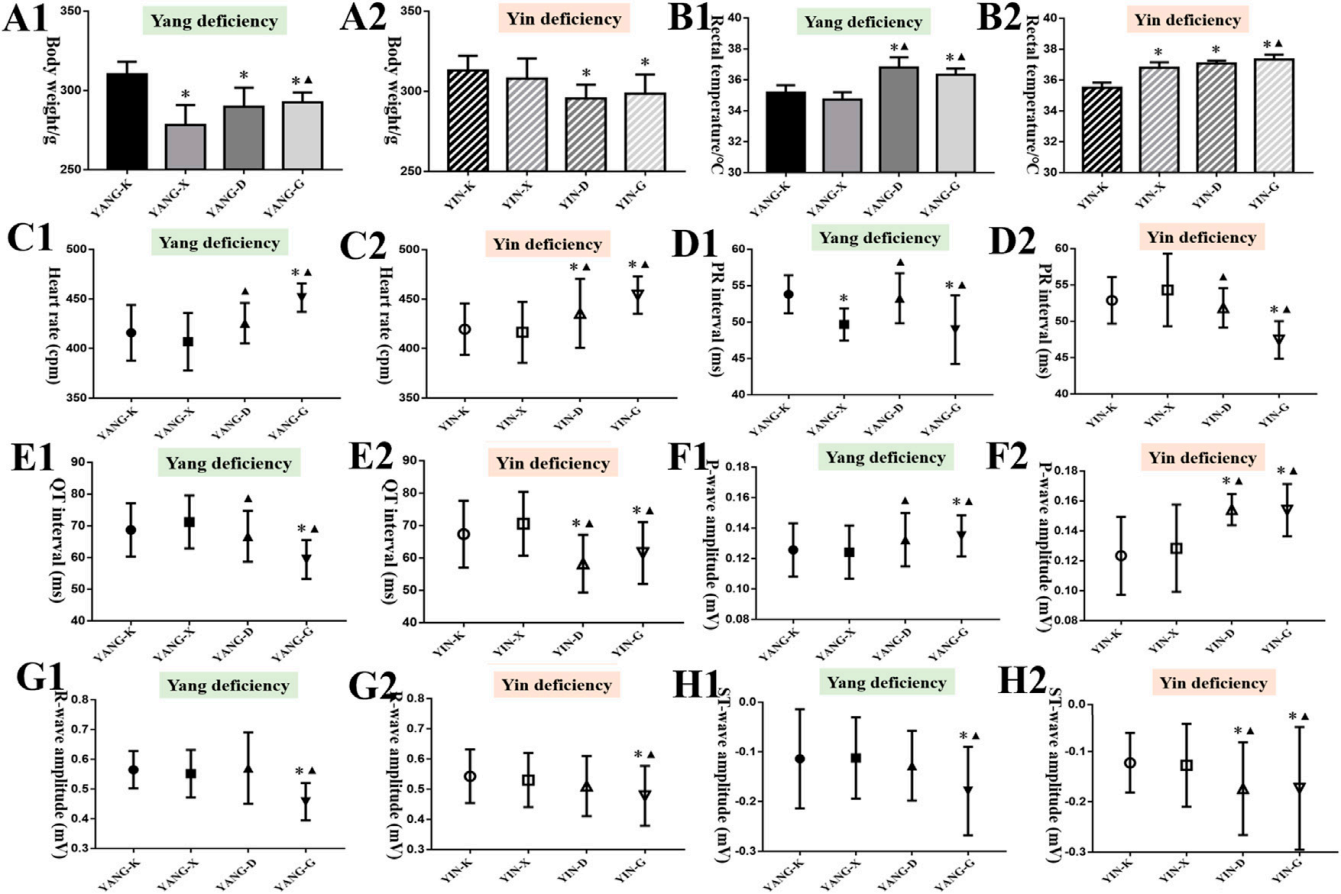
Results of general status and ECG of rats in different groups (14 d). Note: Compared with the corresponding control group, *p < 0.05; compared with the corresponding model group, ^▲^ *p <* 0.05. **(A)** Body weight; **(B)** rectal temperature; **(C)** heart rate; **(D)** PR interval; **(E)** QT interval; **(F)** P-wave amplitude; **(G)** R-wave amplitude; **(H)** ST-wave amplitude.

During the entire experiment *in vivo*, the weight of YANG-X, YANG-D, and YANG-G groups gradually decreased compared to the YANG-K group, while the YANG-D and YANG-G groups’ rectal temperatures increased compared with YANG-K and YANG-X, as presented in Figures 5A,B (*p* < 0.05) (7 days, 14 days, Supplementary Material). In the model of rats with Yin deficiency, there were significant differences in weight and rectal temperature following oral administration of Euodiae Fructus compared with the control group. The changes in general status demonstrate that the treatment of Euodiae Fructus can affect the physical status of rats with Yang or Yin deficiencies, resulting in weight loss and temperature elevation.

#### 3.4.2 ECGs, serum biomarkers, and organ coefficients

It was noticed that long-term exposure to Euodiae Fructus might induce changes in ECG for rats with Yang or Yin deficiency to different degrees. In particular, significant differences in heart rate, PR interval, QT interval, P-wave amplitude, R-wave amplitude, and ST-wave amplitude were observed in the high-dose groups compared with the corresponding control group and model group (Figures 5C–H, Supplementary Material). Namely, long-term and overdose exposure to Euodiae Fructus could cause ECG abnormalities for rats with Yang or Yin deficiencies, including marked prolongation of the ventricular depolarization period and shortening of the effective refractory period, hence disturbing the atrioventricular conduction, which could lead to cardiac arrhythmia.

The results of the alteration in serum myocardial enzymes manifest that the levels of LDH, CK, HBDH, and AST increased in the YANG-G group over the corresponding control group, with a statistically significant difference (*p* < 0.05). Similarly, a remarkable rise of HBDH in the YIN-D group, and LDH, CK, HBDH, and AST in the YIN-G group were also observed over the corresponding control and model groups (*p* < 0.05) (Table 6). Therefore, overdosage and unsuitable syndrome differentiation are associated with the elevation of myocardial enzymes induced by Euodiae Fructus in rats.

**TABLE 6:** Results of serum biomarkers and organ coefficients of rats in different groups (*n* = 8, ‾*x* ± *s*).

Indexes	YANG-K	YANG-X	YANG-D	YANG-G	YIN-K	YIN-X	YIN-D	YIN-G
LDH (U/L)	457.00 ± 89.48	505.88 ± 129.04	490.13 ± 108.98	811.75 ± 164.57*^▲^	466.13 ± 162.89	519.13 ± 107.25	658.25 ± 115.93*	884.50 ± 165.16*^▲^
CK (U/L)	557.90 ± 66.44	571.59 ± 74.52	555.76 ± 74.83	663.79 ± 52.48*^▲^	532.39 ± 79.44	553.26 ± 70.31	606.85 ± 90.18	708.20 ± 127.66*^▲^
HBDH (U/L)	126.74 ± 23.74	138.79 ± 21.86	110.31 ± 13.09	165.03 ± 42.30*	122.59 ± 26.10	122.24 ± 20.18	188.90 ± 43.49*^▲^	203.99 ± 38.23*^▲^
AST (U/L)	113.45 ± 9.47	106.96 ± 5.75	107.91 ± 6.25	130.38 ± 15.93*^▲^	113.33 ± 7.68	116.56 ± 18.24	131.69 ± 17.25	138.66 ± 17.63*^▲^
GLU (mmol/L)	10.28 ± 1.05	7.37 ± 0.98*	7.91 ± 0.67*	9.35 ± 0.83^▲^	9.32 ± 0.68	10.05 ± 0.93	10.05 ± 0.92	10.94 ± 1.11*
TG (mmol/L)	0.57 ± 0.080	0.54 ± 0.070	0.58 ± 0.12	0.68 ± 0.11^▲^	0.52 ± 0.095	0.54 ± 0.083	0.57 ± 0.078	0.65 ± 0.11*
CHO (mmol/L)	1.47 ± 0.094	1.27 ± 0.12*	1.45 ± 0.11^▲^	1.62 ± 0.15^▲^	1.45 ± 0.11	1.61 ± 0.11*	1.66 ± 0.098*	1.68 ± 0.098*
Heart coefficient	0.32 ± 0.012	0.31 ± 0.018	0.31 ± 0.011	0.32 ± 0.0079	0.32 ± 0.016	0.33 ± 0.028	0.34 ± 0.030	0.32 ± 0.0094
Liver coefficient	3.25 ± 0.20	3.30 ± 0.13	3.31 ± 0.14	3.90 ± 0.27*^▲^	3.40 ± 0.19	3.38 ± 0.29	3.57 ± 0.34	4.00 ± 0.15*^▲^
Kidney coefficient	0.40 ± 0.019	0.39 ± 0.027	0.39 ± 0.013	0.39 ± 0.034	0.40 ± 0.019	0.40 ± 0.018	0.41 ± 0.028	0.40 ± 0.025
Lung coefficient	0.47 ± 0.021	0.45 ± 0.034	0.49 ± 0.015	0.47 ± 0.035	0.44 ± 0.026	0.49 ± 0.046	0.50 ± 0.049*	0.50 ± 0.032*
Spleen coefficient	0.31 ± 0.042	0.25 ± 0.056*	0.25 ± 0.022*	0.25 ± 0.037*	0.31 ± 0.030	0.28 ± 0.029	0.26 ± 0.059	0.25 ± 0.039

Note: Compared with the corresponding control group, *p < 0.05; compared with the corresponding model group, ^▲^ *p <* 0.05.

To assess whether Euodiae Fructus involves changes to the glycolipid metabolism of rats with Yang or Yin deficiency, levels of GLU, TG, and CHO were measured in rats exposed to Euodiae Fructus decoction for 14 days. As summarized in Table 6, the high-dose gavage administration for rats with Yang deficiency resulted in significantly changed GLU, TG, and CHO levels compared to the related model groups (*p* < 0.05), while rats with Yin deficiency indicated obvious disorders in GLU, TG, and CHO levels compared to the related control groups (*p* < 0.05). Euodiae Fructus could thus contribute to clinical efficacy for rats with Yang deficiency and metabolic abnormality for those with Yin deficiency.

According to the results of the organ coefficients in Table 6, the obvious differences of heart and kidney were not observed among different groups; however, there was a higher level of liver coefficient in groups of high-dose Euodiae Fructus (*p* < 0.05). The results reveal that an overdose of Euodiae Fructus might contribute to hepatic damage in rats, whether with Yang deficiency or Yin deficiency.

#### 3.4.3 T3 and TSH content in serum, cAMP and cGMP content in plasma, and routine blood test

Aside from changes in glycolipid metabolism, rats with Yang or Yin deficiency also possessed differing content of T3, TSH, cAMP, and cGMP. The level of T3 in the YIN-G group was significantly higher than the corresponding control and model group. Hence, the intervention of Euodiae Fructus could increase cAMP/cGMP in rats with Yin deficiency significantly more than in the related control group (*p* < 0.05) (Table 7). These results suggest that an imbalance of hormone secretion and second messenger transcription might occur due to the irrational usage of Euodiae Fructus.

**TABLE 7:** Results of T3, TSH, cAMP, cGMP, and routine blood test of rats (*n* = 8, ‾*x* ± *s*).

Indexes	YANG-K	YANG-X	YANG-D	YANG-G	YIN-K	YIN-X	YIN-D	YIN-G
T3 (pg/ml)	2.72 ± 0.15	2.69 ± 0.17	2.78 ± 0.093	2.76 ± 0.11	2.75 ± 0.15	2.88 ± 0.15	2.95 ± 0.20*	3.10 ± 0.10*^▲^
TSH (pg/ml)	1.74 ± 0.16	1.86 ± 0.15	1.87 ± 0.15	1.98 ± 0.17*	1.71 ± 0.14	1.64 ± 0.14	0.62 ± 0.12	1.55 ± 0.097
cAMP/cGMP	1.10 ± 0.027	1.06 ± 0.022*	1.06 ± 0.020*	1.08 ± 0.031	1.06 ± 0.015	1.11 ± 0.04*	1.13 ± 0.045*	1.15 ± 0.038*
WBC	6.95 ± 1.61	8.66 ± 1.55	8.61 ± 1.58	11.23 ± 1.77*^▲^	6.53 ± 1.88	10.26 ± 1.08*	10.90 ± 1.89*	12.18 ± 1.31*^▲^
RBC	6.66 ± 0.35	7.34 ± 0.30*	7.26 ± 0.36*	7.41 ± 0.34*	6.93 ± 0.41	7.21 ± 0.71	8.60 ± 0.72*^▲^	9.06 ± 0.59*^▲^
HGB	136.75 ± 6.65	144.63 ± 6.36	142.88 ± 4.46	149.50 ± 7.09*^▲^	138.63 ± 5.31	138.00 ± 5.89	178.50 ± 6.40*^▲^	182.6 ± 8.23*^▲^
PLT	1,172.25 ± 77.72	1,052.5 ± 57.50*	1,151.63 ± 87.76	1,124.25 ± 85.98	1,196.38 ± 46.63	1,168.50 ± 73.97	1,036.50 ± 62.31*^▲^	1,019.75 ± 60.79*^▲^
NEUT%	10.53 ± 1.22	15.97 ± 1.54*	12.89 ± 2.30	25.28 ± 2.73*^▲^	11.05 ± 1.21	11.68 ± 1.38	30.13 ± 3.54*^▲^	34.75 ± 3.13*^▲^
LYMPH%	71.45 ± 4.56	63.03 ± 5.21*	63.08 ± 5.13*	63.43 ± 7.50*	66.08 ± 6.63	61.45 ± 8.26	56.64 ± 8.13	52.83 ± 7.65*
MONO%	5.33 ± 1.32	7.18 ± 1.27*	7.15 ± 1.38*	8.00 ± 1.21*	5.40 ± 0.84	5.33 ± 1.01	5.60 ± 0.70	8.34 ± 0.98*^▲^

Note: Compared with the corresponding control group, *p < 0.05; compared with the corresponding model group, ^▲^ *p <* 0.05.

In addition, the levels of WBC, HGB, and NEUT% in the YANG-G group; of RBC, HGB, PLT, and NEUT% in the YIN-D group; and of WBC, RBC, HGB, PLT, NEUT%, and MONO% in the YIN-G group, were all different from the related model group with a statistically significant difference (*p* < 0.05), indicating continuous gavage with an overdose of Euodiae Fructus for 15 days could influence the blood routine levels of rats with Yang or Yin deficiencies.

#### 3.4.4 Cardiac histology

**FIGURE 6:**
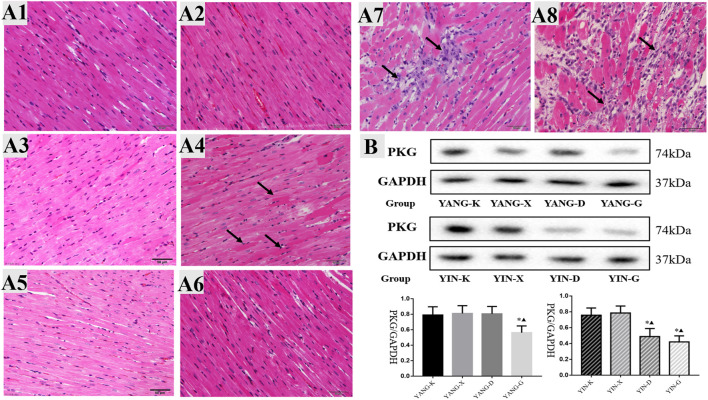
Cardiac histology and protein expression of PKG for rats in different groups. Note: **(A)** cardiac histology and **(B)** protein expression of PKG in heart issue: 1) YANG-K group, 2) YANG-X group, 3) YANG-D group, 4) YANG-G group, 5) YIN-K group, 6) YIN-X group, 7) YIN-D group, and 8) YIN-G group.

As displayed in Figure 6A, obvious histological changes were not observed in the cardiac tissues of the YANG-K group, the YANG-X group, and the YANG-D group, as the cardiac muscle fibers were arranged neatly without inflammatory cell infiltration. In the YANG-G group, some myocardial fiber underwent hypertrophy and became uneven. In the YIN-D group, some cellular edema, break or necrosis, and obvious infiltration of inflammatory cells could be observed. Furthermore, pathological examination revealed that the myocardial fibers were in a disordered condition for the YIN-G group: the major lesions in the myocardial fibers were from degeneration and necrosis, inflammatory infiltration, and edema. These results establish that cardiac pathological injury in rats is associated with overdosage and unsuitable syndrome differentiation of Euodiae Fructus.

#### 3.4.5 Protein expression of PKG in heart issue

The inhibitory effects of Euodiae Fructus for the protein expression of PKG were concentration-dependent in rats with Yin deficiency, while the protein expression of PKG in heart issue was also lower in the YANG-G group than in the corresponding control and model groups, and statistically significant differences were observed among these groups (Figure 6B, Supplementary Material).

### 3.5 Untargeted metabolomics of cardiotoxicity induced by Euodiae Fructus in rats

#### 3.5.1 Multivariate data analysis

**FIGURE 7:**
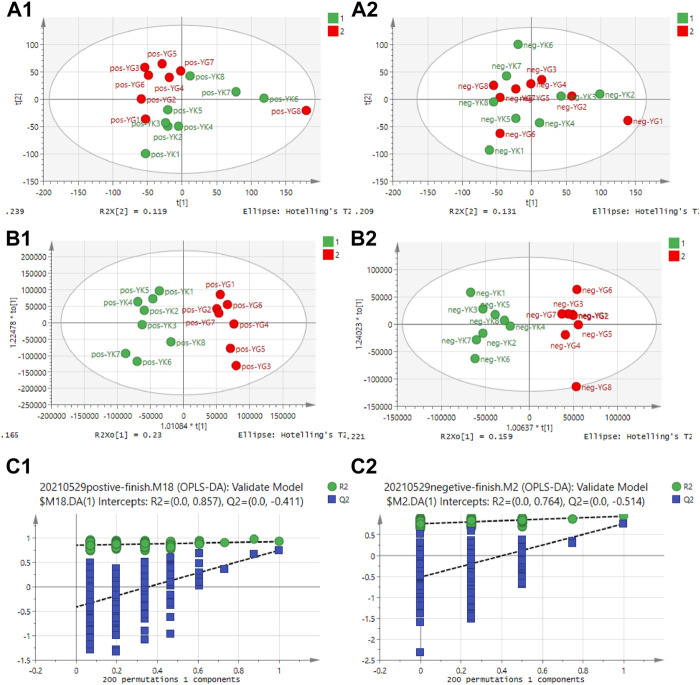
Results of multivariate data analysis for rats in YIN-K and YIN-G groups. Note: **(A1)** PCA scores plot-ESI^+^; **(A2)** PCA scores plot-ESI^−^; **(B1)** OPLS-DA scores plot-ESI^+^; **(B2)** OPLS-DAscores plot-ESI^−^; **(C1)** Permutation plot-ESI^+^; **(C2)** Permutation-scores plot-ESI^−^.

The untargeted metabolomics of cardiotoxicity induced by Euodiae Fructus in rats with Yin deficiency were evaluated; the serum samples of the YIN-K and YIN-G groups were determined using UHPLC-Q-Exactive Orbitrap/MS. According to the results of the multivariate data analysis in Figure 7, there was clear separation between the YIN-K and YIN-G groups, suggesting the metabolic profile might be different after continuous gavage of Euodiae Fructus for 15 days, and the details of PCA, OPLS-DA, and permutations are shown in the Supplementary Material.

#### 3.5.2 Metabolites analysis

Based on the limitation of the variables with VIP>1 and simultaneous significant difference, ultimately there were 3212 endogenous metabolites in total, with 2060 (64.13%) in the positive ion mode, and the remaining in the negative mode (35.87%). After the identification, 34 corresponding metabolites were highlighted as the most discriminant in the rats of the YIN-K and YIN-G groups, including D-proline, deoxycytidine, 5-hydroxyisourate, cytosine, uric acid, D-lysine, and so on (Supplementary Material).

**FIGURE 8:**
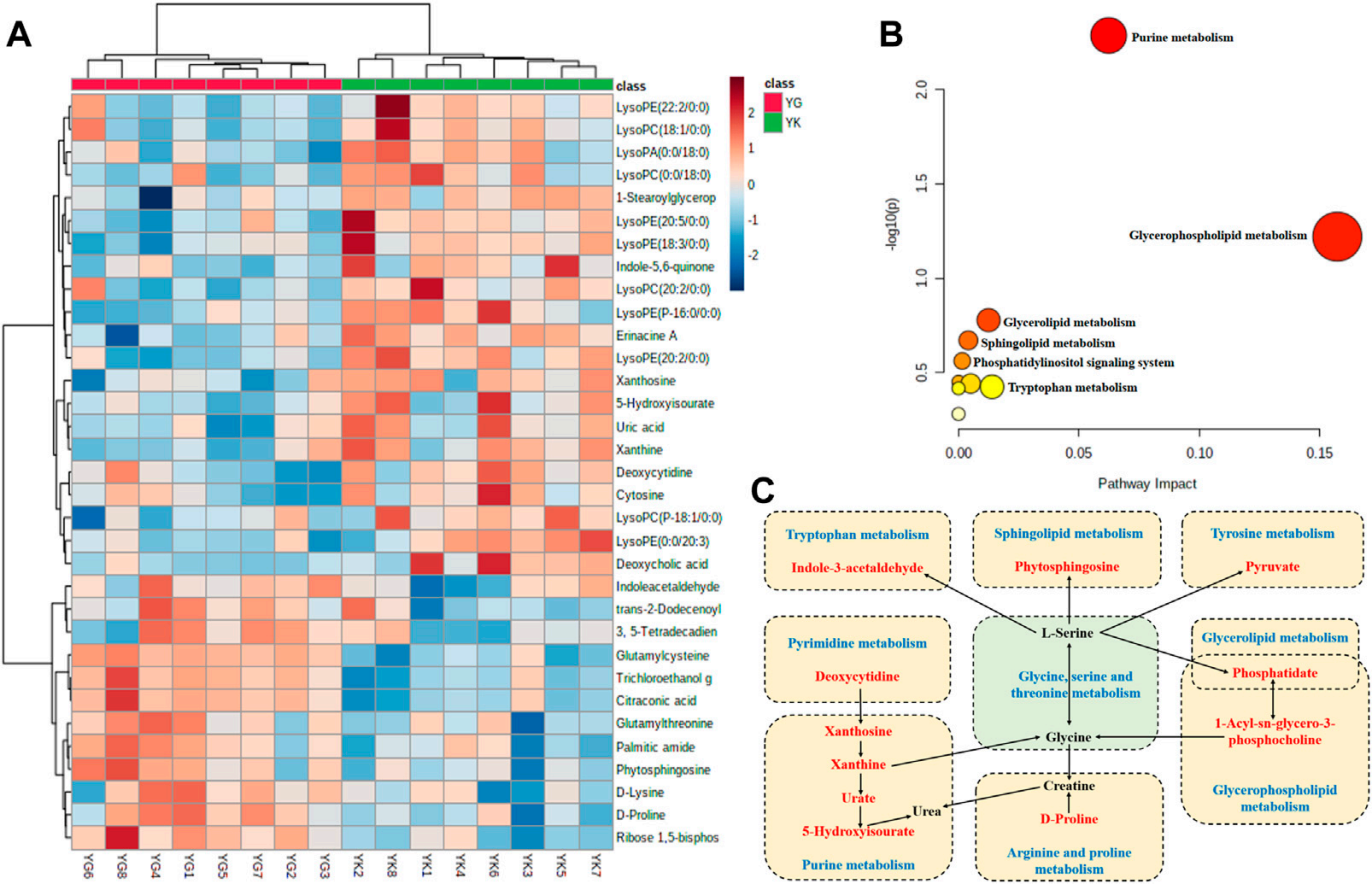
Metabolites analysis of rat serum samples in YIN-K and YIN-G groups. Note: **(A)** cluster analysis; **(B)** pathway analysis; **(C)** summary of metabolites and pathways (blue words indicate metabolic pathways, red words indicate identified discriminant metabolites in present research, and black words indicate the related endogenous metabolites).

The cluster analysis depicted in Figure 8A reveals that these discriminant metabolites were divided into two categories in a dendrogram, and there was close correlations or similar pathways for the metabolites in the same category. Furthermore, the results of the pathway analysis also pointed out that 10 metabolic pathways, including the purine metabolism, glycerophospholipid metabolism, glycerolipid metabolism, sphingolipid metabolism, and the phosphatidylinositol signaling system, as well as the arginine and proline metabolisms, were all strongly involved in the metabonomic signatures of rats exposed to Euodiae Fructus. This could induce cardiotoxicity in rats with Yin deficiency, and the most likely metabolic pathways and related discriminant metabolites are exhibited in Figures 8B,C and in the Supplementary Material.

## 4 Discussion

In recent decades, the therapeutic and beneficial effects of Chinese *materia medica* in preventing or ameliorating multiple cardiovascular and chronic diseases have become increasingly well known. Correspondingly, public awareness of medicinal herb safety has also heightened (Amadi and Orisakwe, 2018; Shaito et al., 2020). The present findings from *in vivo* and *in vitro* experiments and untargeted metabolomics research reveal that the mechanisms of potential cardiotoxicity induced by overdosage and irrational usage of Euodiae Fructus involve the cGMP-PKG pathway and the metabolic pathways concerned with energy metabolism, lipid metabolism, oxidative stress, and so on.

### 4.1 Cardiac cytotoxicity of evodiamine and rutaecarpine in *in vivo* experiments

The cGMP-PKG pathway has been closely linked with the cardiac cytotoxicity of evodiamine and rutaecarpine. Our data suggest the levels of LDH and CK, and the mitochondrial membrane potential and intensity of calcium fluorescence, changed remarkably in H9c2 cells undergoing the administration of evodiamine and rutaecarpine, which was similar to ways in which NRCMs shared frequency of spontaneous beat.

It is accepted that determination of LDH and CK activity provides one of the biochemical indexes for the evaluation and diagnosis of heart disease, due to the level of LDH in serum reflecting injury in the permeability of the cardiomyocytes, and the activity level of CK being directly related to the consumption and supply of myocardial oxygen and energy, muscle contraction, and mitochondrial function (Agress, 1965; Matschke et al., 2005; Ingwall, 2009; Zervou et al., 2016; Bak and Schousboe, 2017; Klein et al., 2020). Furthermore, the mitochondrial membrane potential and the intensity of calcium fluorescence, which were testing indexes in the present study, play an essential role in the mitochondrial function of myocardial tissue homeostasis (Skarka and Ostadal, 2002; Dibb et al., 2007; Kadenbach et al., 2011; Davlouros et al., 2016; Zorova et al., 2018; Schartner et al., 2019; Lai and Qiu, 2020). Understanding of the electrophysiological effects in cardiomyocyte contractile and mechanical function in response to cardiotoxic drugs has previously relied on primary cardiomyocytes from animal models (Liu et al., 2012; Tang et al., 2016; Blair and Pruitt, 2020). Therefore, this research selected the abovementioned indexes to quantify the myocardial cytotoxicity of evodiamine and rutaecarpine in an effort to understand how these bioactive compounds of Euodiae Fructus directly impact the cGMP-PKG pathway at the cellular and cardiomyocytes levels. Although there are empirical studies emphasizing the cardiovascular protective effects of evodiamine and rutaecarpine (Jiang et al., 2017; Zeng et al., 2019), some researchers have verified the risk of arrhythmia and cardiotoxicity *in vivo* and *in vitro*, findings consistent with the results of our study. For example, depending on dosage, dehydroevodiamine and hortiamine could prolong the action potential duration, eventually resulting in proarrhythmic effects (Baburin et al., 2018).

The cGMP-PKG signaling pathway plays a crucial role in various myocardial pathophysiological process, including cell growth and survival, interstitial fibrosis, endothelial permeability, myocardial contraction, and cardiovascular remodeling (Inserte and Garcia-Dorado, 2015; Nakamura and Tsujita, 2021). In particular, the cGMP-PKG pathway is a principal factor implicated in cardiovascular complications of diverse etiological processes because it stimulates downstream targets, including the Ca^2+^ channel, and a β3-adrenceptor in an inhibitory G protein-dependent manner (Takimoto, 2012; Zhang et al., 2014; Arioglu-Inan et al., 2019; Wan et al., 2020). With growing recognition of the cGMP-PKG pathway, there is increasing interest in envisaging it as a therapeutic target against the cardiotoxic effects of some drugs. Cumulatively and progressively developing the cardiomyopathy caused by adriamycin, levosimendan and tadalafil could produce greater benefits of anti-cardiotoxicity and prevent cardiomyocyte apoptosis by activating the cGMP-PKG pathway (Koka et al., 2010; Efentakis et al., 2020). Interference with hypotension and bradycardia among the molecular and cellular determinants of the cardiotoxicity induced by Crotalusdurissus cascavella venom, contributing to negative inotropic effects of the heart, have been associated with the NO/cGMP/PKG pathway (Simoes et al., 2021). Understanding the key role of the cGMP-PKG pathway in the cardiac cytotoxicity of evodiamine and rutaecarpine is essential for reducing risk in the clinical usage of Euodiae Fructus, and present research confirms the related mechanism through the agonist of the PKG protein, the PKG drug G1, as well as the following *in vitro* experiments and untargeted metabolomics research.

### 4.2 Cardiotoxicity induced by Euodiae Fructus in *in vitro* experiments

In general, the quintessence of TCM is syndrome differentiation and treatment, and the guarantee of clinical efficacy is the safe use of medications (Shaw et al., 2012; Xiang et al., 2019). Euodiae Fructus is considered slightly poisonous with hot or warm properties, and is used for treating gastro-intestinal disorders belonging to Yang deficiency in the theory of traditional Chinese medicine (TCM) (Chinese Pharmacopoeia Commission, 2020; Li et al., 2020). Clinical medication factors are complex in practice; the overdosage and irrational usage of Euodiae Fructus are cause for concern because some cases are associated with serious heart disorders and deaths. Accordingly, the current research illustrates the potential cardiotoxicity induced by Euodiae Fructus, and the results in rats with Yin deficiency suggest obvious cardiac physiological function, abnormal ECG, and pathological injury in the high-dosage group of Euodiae Fructus.

First, in order to further explore the clinical problems and simulate clinical symptoms, our study effected a hydrocortisone-induced Yang deficiency and a thyroxine-induced Yin deficiency model in rats, with the relevant modeling methods having certain recognition in syndrome animal modeling under TCM theoretical guidelines (Yao et al., 2007; Han et al., 2013; Ling and Xu, 2013; Reheman et al., 2019; Hu et al., 2021). Notably, the overall characterization, involving the general state, body weight, body temperature, and organ coefficients, in combination with the levels of T3 and TSH in serum, cAMP/cGMP in plasma, and glucose and lipid metabolism were comprehensively evaluated in our experiments.

Second, the transformation of “health benefit” into “cardiac toxicity” for Euodiae Fructus in terms of different syndromes and dosages was investigated based on ECG readings, serum myocardial zymogram results, and cardiac histology. The ECG was foundational in assessing cardiac function in terms of rate and rhythm, and is universally available for the diagnosis of heart diseases (Klabunde, 2017; Teplitzky et al., 2020). Moreover, the determination of cardiac enzyme profiles, including CK, CK-MB, HBDH, LDH, and AST, as evidence of myocardial injury, has been confirmed by substantial research, such as those studies exploring myocardial ischemic necrosis or changes to membrane permeability in myocardial cells (Pappas, 1989; Lee et al., 2009). Despite some promising biomedical approaches in the cardiac research field, cardiac histology is still irreplaceable in the diagnosis of cardiac injuries, owning to the ability of cardiac tissue slices to provide details of the native multicellularity, architecture, and physiology of the heart (Watson et al., 2019; George et al., 2020; Perbellini and Thum, 2020). In our study, an overdose of Euodiae Fructus could induce cardiotoxicity for rats in a state of Yin deficiency, including abnormal ECG and myocardial enzyme results, and cardiac pathological injuries, suggesting that irrational usage and overdosage of Euodiae Fructus is associated with increased risk of potential cardiotoxicity. Our study thus adequately identifies the urgent need to develop pharmacovigilance practices for herbal medicines, to monitor the cardiac function of patients, and to standardize clinical medication to avoid related adverse reactions (Barnes, 2003; Wang et al., 2009).

### 4.3 The interpretation of untargeted metabolomics research

As the terminal of an organism’s biological process, an altered metabolism is one of the hallmarks of noxious effects in the heart, where changes in protein expression and injures in cardiac function ultimately lead to aberrant cellular metabolism (Kroemer et al., 2018; Luz and Tokar, 2018). Fortunately, the emergence of metabolomics research has provided a new approach to statistically and quantitatively visualizing evidence according to the dynamic information in overall profiles of endogenous metabolites after the biological system has suffered from exogenous disturbance and stimulation (Al-Ansari et al., 2021; Shibutami and Takebayashi, 2021; Spyroglou et al., 2021). Indeed, a burst of research utilizing untargeted metabolomics technology has been published in the field of cardiac toxicology over the past decades, based on the dual advantages of global material scanning and the accuracy of material annotation, and contributing to numerous methodological advances in interpreting the enrolled metabolic pathway and toxic mechanism (Parry et al., 2018; Palmer et al., 2020). Here, the methods of untargeted metabolomics research and multivariate statistics were used to detect changes in endogenous metabolisms induced by overdosage of Euodiae Fructus in rats with Yin deficiency. Our result highlights 34 kinds of metabolites, including D-proline, deoxycytidine, 5-hydroxyisourate, cytosine, uric acid, and D-lysine, and a total of 10 metabolic pathways involving the purine metabolism, glycerophospholipid metabolism, glycerolipid metabolism, sphingolipid metabolism, and the phosphatidylinositol signaling system, as well as the arginine and proline metabolisms.

On the one hand, through investigation of potential molecular mechanisms underlying different conditions in biological systems, the expression patterns of some differential metabolites were similar, due to involving the associated metabolic pathways, resulting in the presentation of a close concentration-dependent correlation (You et al., 2019; Jahagirdar and Saccenti, 2020). In this study, the levels of lysophospholipids (lysophosphatidic acid, lysophosphatidylcholine) and lysosphingolipids, namely LysoPC (18:1/0:0), LysoPC (0:0/18:0), LysoPE (22:2/0:0), and LysoPA (0:0/18:0), decreased in the YIN-G group, suggesting that in a Yin deficiency state, high-dose Euodiae Fructus can reduce the lysophosphatidic content and cause possible heart risk. According to published research, LPC (14:0) and LPC (20:2) were verified as highly specific biomarkers of cardiotoxicity from rat plasma samples *via* ultra-performance liquid chromatography quadrupole time-of-flight mass spectrometry, and subsequently used a support vector machine to develop a predictive model (Li et al., 2015). As a critical biomarker positively associated with cardiovascular issues, there is increasing evidence showing that lysophospholipids and lysosphingolipids can specifically bind to G-protein coupled receptors, thus directly control secondary messengers involving the Ca^2+^ signaling pathway, Rho Kinase (ROCK), diacylglycerol (DAG), IP3 receptor (IP3R), mitogen-activated protein kinase (MAPK), adenylate cyclase (AC), and phosphatidylinositol 3-kinase (PI3K), etc. (Schilling et al., 2002; Torkhovskaya et al., 2007; Li et al., 2016; Law et al., 2019). Hence, the regulation of lysophospholipids on the G-coupled protein and calcium pathway is similar to the expression level and regulatory function of cGMP-PKG pathway involved in this study.

On the other hand, the cardiotoxicity induced by overdosage and irrational usage of Euodiae Fructus is associated with the purine metabolism, glycerophospholipid metabolism, glycerolipid metabolism, and the sphingolipid metabolism, as well as the phosphatidylinositol signaling system, suggesting that the related cardiotoxic metabolic pathways could mediate oxidative stress, energy metabolism, lipid metabolism, amino acid metabolism and other biological processes. With regard to the purine metabolism in cardiac pathological process, findings demonstrate overwhelmingly that purine release is directly related to the rate of energy consumption in the heart, and is significantly connected to a wide range of cardiovascular activity, including dilating the coronary artery, reducing reperfusion injury, inhibiting cardiomyocyte apoptosis, and so on. Furthermore, this metabolic pathway is involved in the oxidative stress injury of cardiomyocytes caused by the release of reactive oxygen species (Hisatome et al., 1990; Zucchi et al., 1990; Safranow et al., 2005; Sansbury et al., 2014). The sphingolipids are also known to play a pivotal role in signal transduction; growth and differentiation; immune response, proliferation, and apoptosis; inflammatory response; and other important signal molecules. Sphingolipid metabolism disorder has been widely identified as a prognostic and diagnostic marker for cardiovascular diseases, such as ischemia-reperfusion injury, lipotoxic cardiomyopathy, and cardiac insufficiency in recent lipomics studies, while some specific sphingolipids are new biomarkers for cardiovascular diseases (Baranowski and Gorski, 2011; Iqbal et al., 2017; Hannun and Obeid, 2018; Matanes et al., 2019; Iessi et al., 2020).

## 5 Conclusion

To the best of our knowledge, no previous study has specifically focused on the mechanisms of potential cardiotoxicity induced by Euodiae Fructus. The present research can thus provide a useful overview of how overdosage and irrational usage of Euodiae Fructus can induce cardiac side effects at macroscopic and microscopic levels, including the organism, tissue, cell, protein, and molecular levels, and hence what needs to be done to improve the safety of herbal medicines, especially herbs with poisonous components. Inevitably, this study is only a preliminary investigation into the cardiac cytotoxicity of evodiamine and rutaecarpine through *in vivo* experiments, and into the expression of the cGMP-PKG pathway in discussions of the differential metabolites in rat serum. Based on our data, it is clear that further research needs to be performed using mass spectrometry and gas chromatography to detect and analyze tissue samples, such as those of myocardium, liver, and kidney, so as to fully tap the overall metabolomic information. Further investigations are warranted to explore the cardiotoxicity profiles and other toxicity correlations of Euodiae Fructus, as well as its toxic ingredients.

## Data Availability

The original contributions presented in the study are included in the article/Supplementary Material, and further inquiries can be directed to the corresponding author.
