# Supplementary material for: Potential cardiotoxicity induced by Euodiae Fructus: In vivo and in vitro experiments and untargeted metabolomics research
Source: Front Pharmacol. 2022 Oct 24;13:1028046. doi: 10.3389/fphar.2022.1028046 (PMC9637925; doi:10.3389/fphar.2022.1028046)
Supplement: Supplementary file 1 [file DataSheet1.docx]

Supplementary Material

# The protein expression of cGMP-PKG pathway of evodiamine and rutaecarpine

**Table S1.** The protein expression of cGMP-PKG pathway (*n* = 3, ‾*x* ± *s*)

| **Groups** | **Concentration (μmol/L)** | **cGMP/GAPDH** | **PKG/GAPDH** |
| --- | --- | --- | --- |
| Evodiamine | 0 | 0.70 ± 0.048 | 0.66 ± 0.092 |
|  | 5 | 0.40 ± 0.039** | 0.46 ± 0.057* |
|  | 10 | 0.37 ± 0.052** | 0.35 ± 0.069** |
|  | 25 | 0.17 ± 0.039** | 0.24 ± 0.049** |
| Rutaecarpine | 0 | 0.66 ± 0.10 | 0.32 ± 0.032 |
|  | 60 | 0.42 ± 0.016* | 0.29 ± 0.021 |
|  | 80 | 0.38 ± 0.088* | 0.17 ± 0.039** |
|  | 100 | 0.22 ± 0.064** | 0.15 ± 0.036** |

Note: Compared with the control group, **P* < 0.05, ***P* < 0.01.


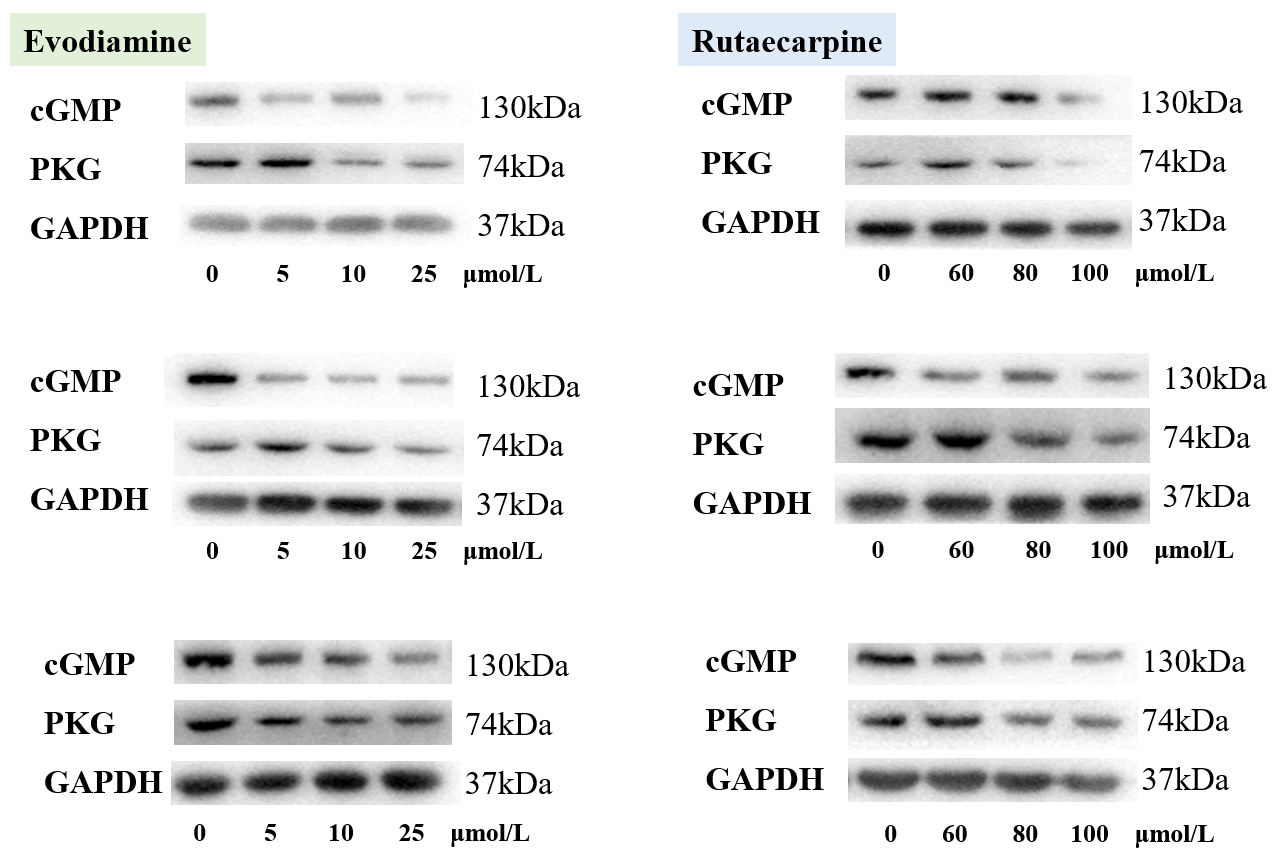


**Figure S1.** The protein expression of cGMP-PKG pathway of evodiamine and rutaecarpine.

# Cell viability and cytotoxicity assay of evodiamine and rutaecarpine plus PKG drug G1

**Table S2.** Cell viability assay and leakage of LDH of evodiamine and rutaecarpine plus PKG drug G1 (*n* = 6, ‾*x* ± *s*)

| **Groups** | **Concentration (μmol/L)** | **Cell viability (%)** | **Leakage of LDH (%)** |
| --- | --- | --- | --- |
| Evodiamine | 0 | 100.00 ± 6.47 | 7.09 ± 0.30 |
|  | P | 98.41 ± 9.19 | 9.67 ± 1.68 |
|  | 5 | 57.30 ± 10.98*^▲^ | 9.13 ± 0.23 |
|  | 5+P | 63.14 ± 10.60*^▲^ | 8.08 ± 0.64^#^ |
|  | 10 | 48.23 ± 5.85*^▲^ | 11.21 ± 1.38* |
|  | 10+P | 54.91 ± 5.65*^▲^ | 8.53 ± 1.69 |
|  | 25 | 41.32 ± 5.56*^▲^ | 24.11 ± 1.68*^▲^ |
|  | 25+P | 47.38 ± 7.21*^▲^ | 23.39 ± 3.40*^▲^ |
| Rutaecarpine | 0 | 100.00 ± 7.24 | 6.73 ± 0.36 |
|  | P | 89.55 ± 9.82 | 8.50 ± 1.33 |
|  | 60 | 68.81 ± 7.29*^▲^ | 14.64 ± 1.46*^▲^ |
|  | 60+P | 74.15 ± 8.60* | 11.50 ± 1.40*^#^ |
|  | 80 | 55.92 ± 7.39*^▲^ | 14.50 ± 0.96*^▲^ |
|  | 80+P | 59.38 ± 1.95*^▲^ | 12.27 ± 1.38*^▲^ |
|  | 100 | 46.41 ± 6.94*^▲^ | 19.03 ± 1.31*^▲^ |
|  | 100+P | 52.13 ± 8.47*^▲^ | 16.19 ± 2.14*^▲^ |

Note: Compared with the control group (non-medication), **P <* 0.05; compared with PKG drug G1 group, ^▲^*P <* 0.05; compared with single compound group (corresponding dose), ^#^*P <* 0.05: P represented 5 μmol/L PKG drug G1.

# The protein expression of PKG of evodiamine and rutaecarpine plus PKG drug G1

**Table S3.** The protein expression of PKG of evodiamine and rutaecarpine plus PKG drug G1 (*n* = 3, ‾*x* ± *s*)

| **Groups** | **Concentration (μmol/L)** | **PKG/GAPDH** | **Groups** | **Concentration (μmol/L)** | **PKG/GAPDH** |
| --- | --- | --- | --- | --- | --- |
| Evodiamine | 0 | 0.65 ± 0.072 | Rutaecarpine | 0 | 0.57 ± 0.044 |
|  | P | 0.70 ± 0.091 |  | P | 0.68 ± 0.13 |
|  | 5 | 0.36 ± 0.089*^▲^ |  | 60 | 0.49 ± 0.093 |
|  | 5+P | 0.49 ± 0.026^▲^ |  | 60+P | 0.64 ± 0.04 |
|  | 10 | 0.54 ± 0.053 |  | 80 | 0.36 ± 0.064^▲^ |
|  | 10+P | 0.58 ± 0.031 |  | 80+P | 0.56 ± 0.085^#^ |
|  | 25 | 0.24 ± 0.053*^▲^ |  | 100 | 0.26 ± 0.085*^▲^ |
|  | 25+P | 0.15 ± 0.049*^▲^ |  | 100+P | 0.20 ± 0.016*^▲^ |

Note: Compared with the control group (non-medication), **P <* 0.05; compared with PKG drug G1 group, ^▲^*P <* 0.05; compared with single compound group (corresponding dose), ^#^*P <* 0.05: P represented 5 μmol/L PKG drug G1.


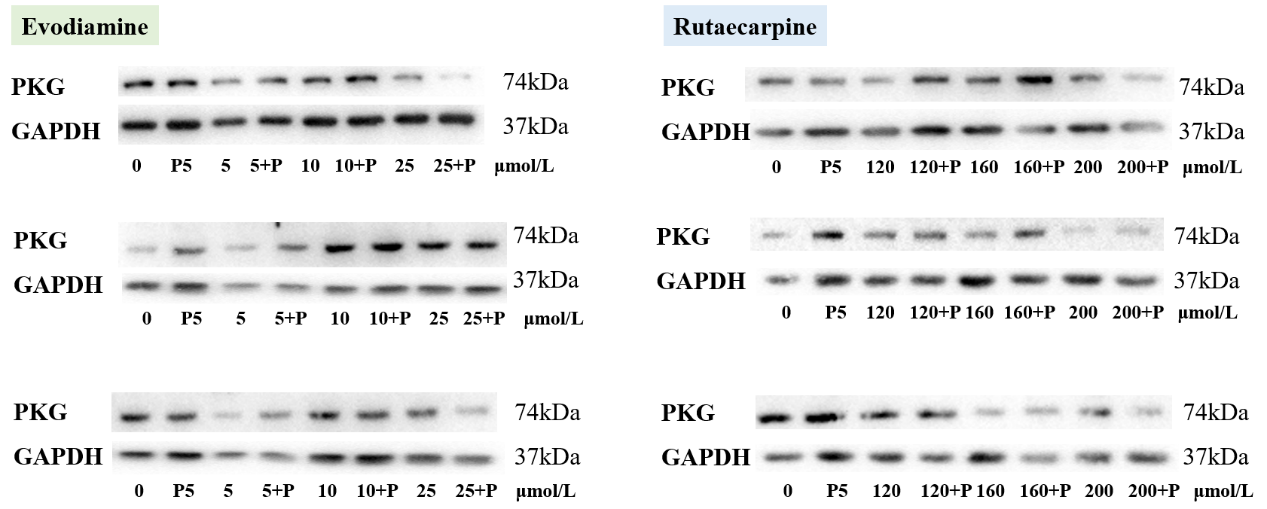


**Figure S2.** The protein expression of PKG of evodiamine and rutaecarpine plus PKG drug G1.

# The body weight and rectal temperature of rats in different groups

**Table S4.** The body weight and rectal temperature of rats in different groups (*n* = 8, ‾*x* ± *s*)

| **Body weight/g** | | | **Rectal temperature/℃** | |
| --- | --- | --- | --- | --- |
| **Groups** | **7 d** | **14 d** | **7 d** | **14 d** |
| YANG-K | 251.58 ± 9.16 | 310.13 ± 7.42 | 37.23 ± 0.78 | 35.18 ± 0.44 |
| YANG-X | 243.00 ± 14.50 | 278.25 ± 11.65* | 36.98 ± 0.81 | 34.71 ± 0.45 |
| YANG-D | 239.55 ± 13.23 | 289.80 ± 11.17* | 37.10 ± 0.66 | 36.80 ± 0.60*^▲^ |
| YANG-G | 237.90 ± 11.04* | 292.50 ± 5.79*^▲^ | 37.28 ± 0.83 | 36.31 ± 0.38*^▲^ |
| YIN-K | 253.50 ± 14.11 | 312.90 ± 8.72 | 37.14 ± 0.97 | 35.49 ± 0.318 |
| YIN-X | 255.67 ± 13.46 | 307.88 ± 11.87 | 37.28 ± 0.53 | 36.80 ± 0.32* |
| YIN-D | 252.08 ± 10.84 | 295.38 ± 8.21* | 37.21 ± 0.56 | 37.08 ± 0.17* |
| YIN-G | 243.17 ± 10.07^▲^ | 298.40 ± 11.41* | 37.36 ± 0.74 | 37.31 ± 0.31*^▲^ |

Note: Compared with the corresponding control group, **P <* 0.05; compared with the corresponding model group, ^▲^*P <* 0.05.

# The results of ECG of rats in different groups

**Table S5.** The results of ECG of rats in different groups (*n* = 8, ‾*x* ± *s*)

| **ECG** | **YANG-K** | **YANG-X** | **YANG-D** | **YANG-G** | **YIN-K** | **YIN-X** | **YIN-D** | **YIN-G** |
| --- | --- | --- | --- | --- | --- | --- | --- | --- |
| Heart rate(cpm) | 415.88± 27.73 | 406.92± 28.66 | 425.58± 20.19^▲^ | 451.29± 14.18*^▲^ | 419.60± 25.80 | 416.40± 30.53 | 435.60± 34.61*^▲^ | 454.06± 18.77*^▲^ |
| PR interval(ms) | 53.81± 2.60 | 49.67± 2.18* | 53.27± 3.41^▲^ | 48.94± 4.68* | 52.88± 3.16 | 54.31± 4.93 | 51.83± 2.68^▲^ | 47.44± 2.55*^▲^ |
| QT interval(ms) | 68.67± 8.37 | 71.21± 8.27 | 66.67± 7.94 | 59.35± 6.08*^▲^ | 67.31± 10.23 | 70.54± 9.74 | 58.21± 8.79*^▲^ | 61.48± 9.43*^▲^ |
| P-wave amplitude(mV) | 0.13± 0.017 | 0.12± 0.017 | 0.13± 0.017^▲^ | 0.13± 0.013*^▲^ | 0.12± 0.026 | 0.13± 0.029 | 0.15± 0.010*^▲^ | 0.15± 0.017*^▲^ |
| R-wave amplitude(mV) | 0.56± 0.062 | 0.55± 0.079 | 0.57± 0.12 | 0.46± 0.062*^▲^ | 0.54± 0.088 | 0.53± 0.089 | 0.51± 0.098 | 0.48± 0.098*^▲^ |
| ST-wave amplitude(mV) | -0.11± 0.099 | -0.11± 0.081 | -0.13± 0.037 | -0.18± 0.056*^▲^ | -0.12± 0.024 | -0.13± 0.034 | -0.17± 0.050*^▲^ | -0.17± 0.099*^▲^ |

Note: Compared with the corresponding control group, **P <* 0.05; compared with the corresponding model group, ^▲^*P <* 0.05.

(1) YANG-K group


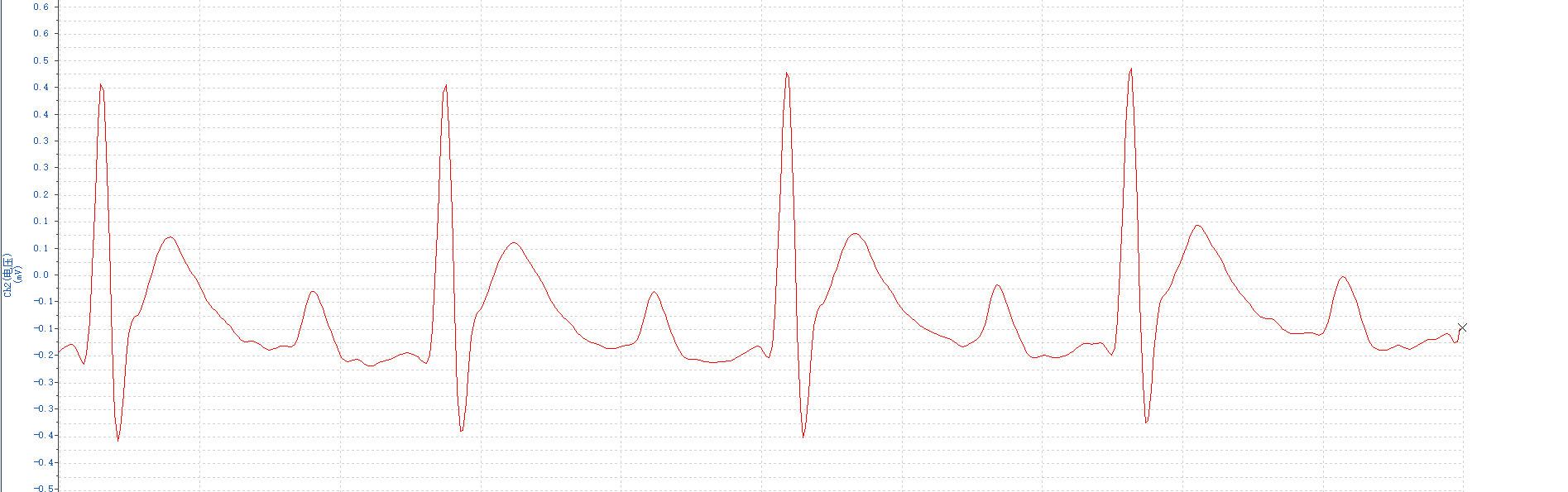


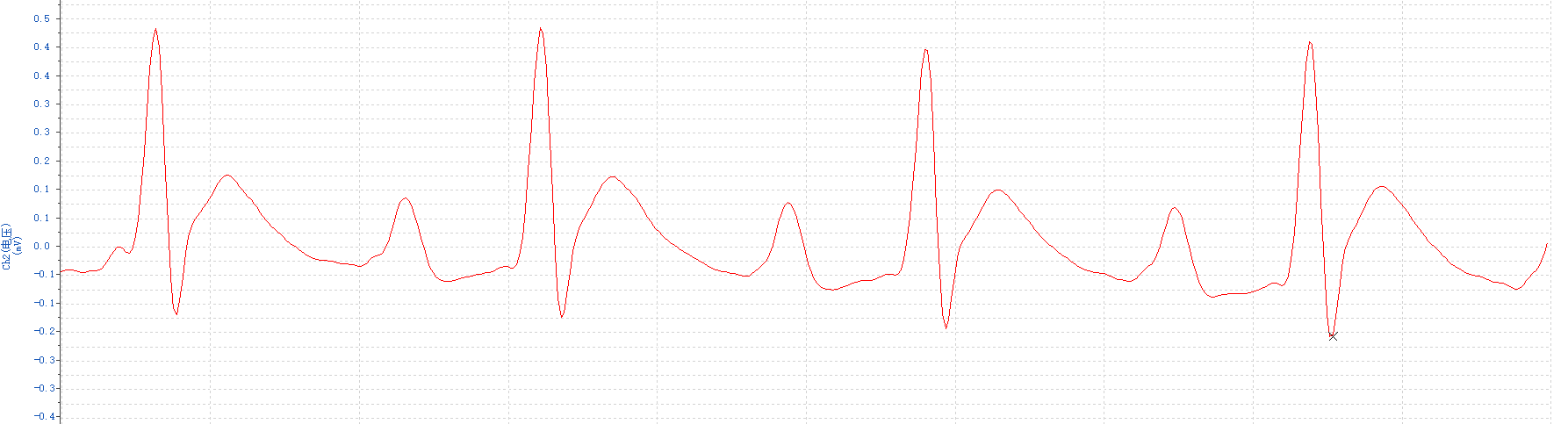


(2) YANG-X group


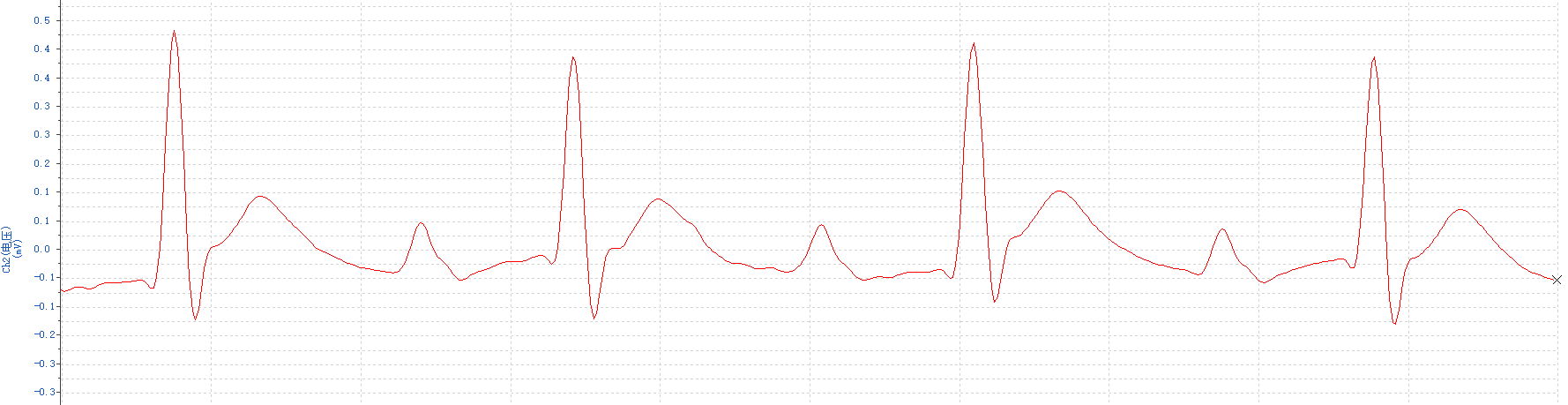


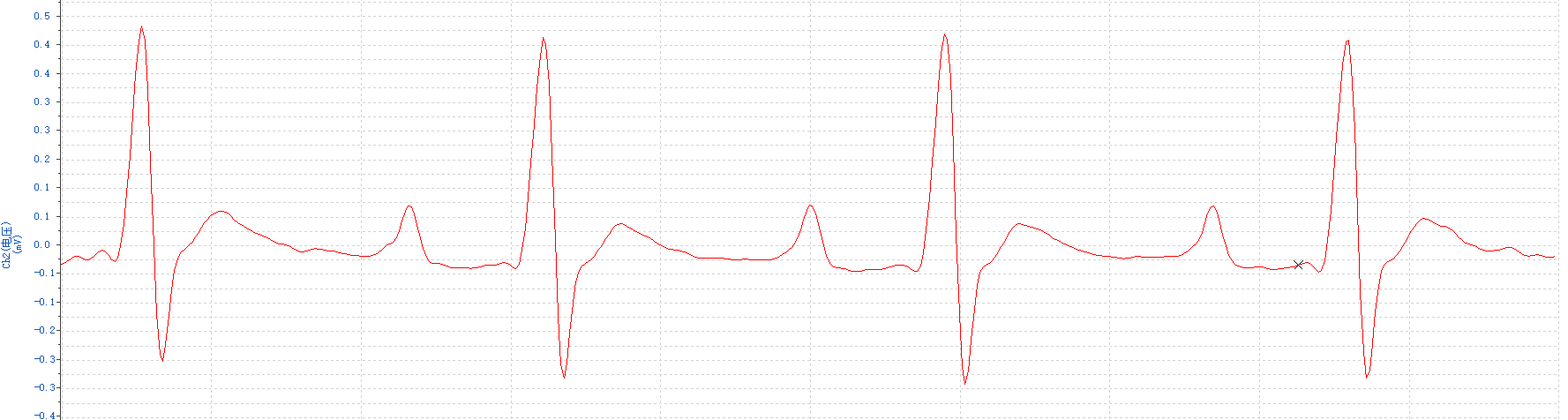


(3) YANG-D group


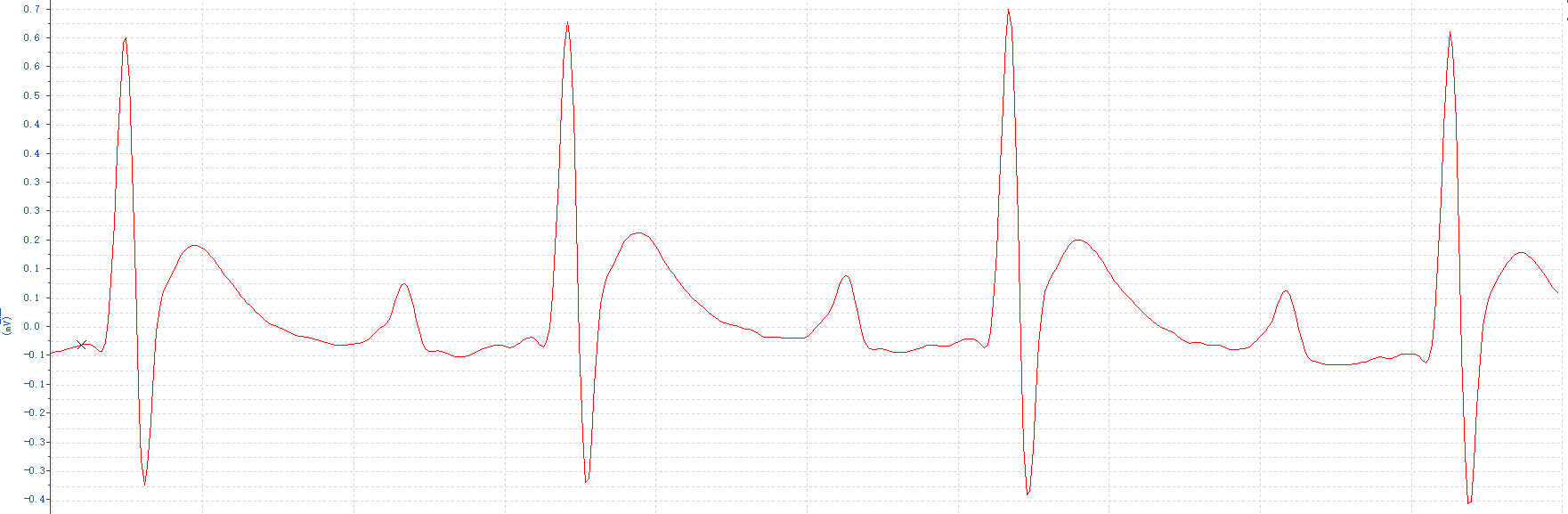


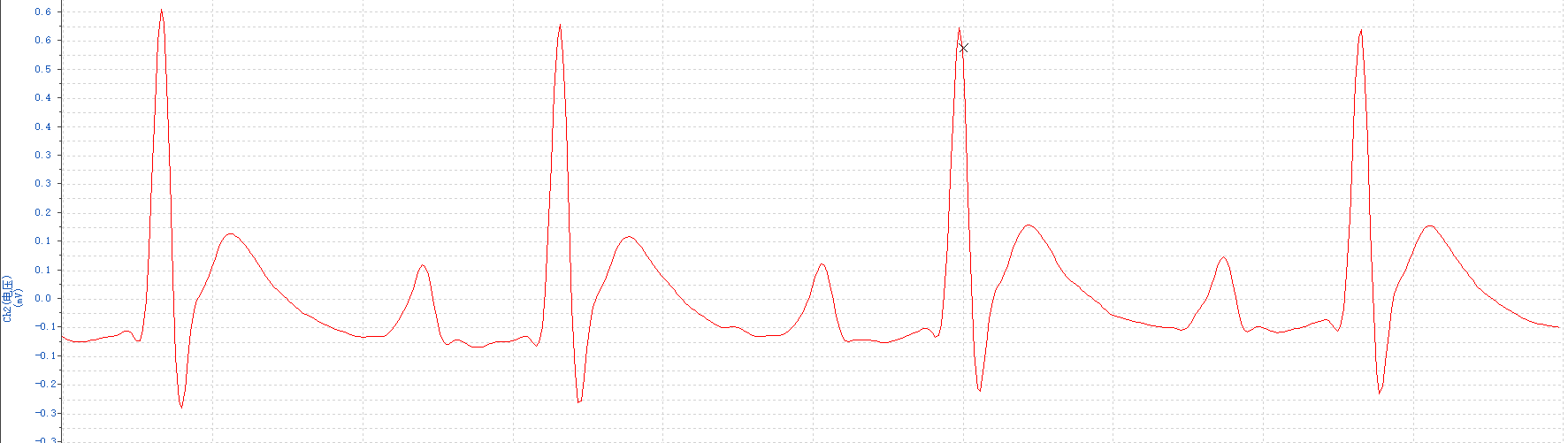


(4) YANG-G group


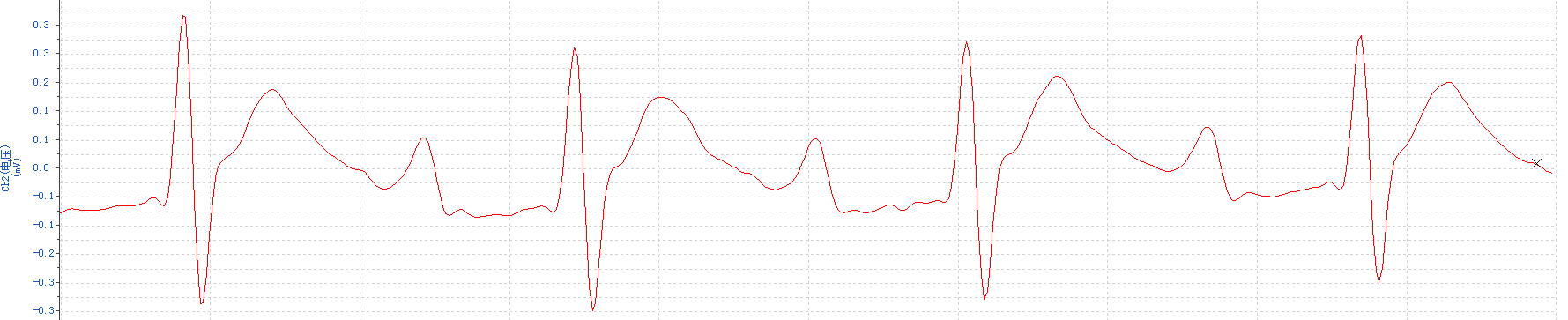


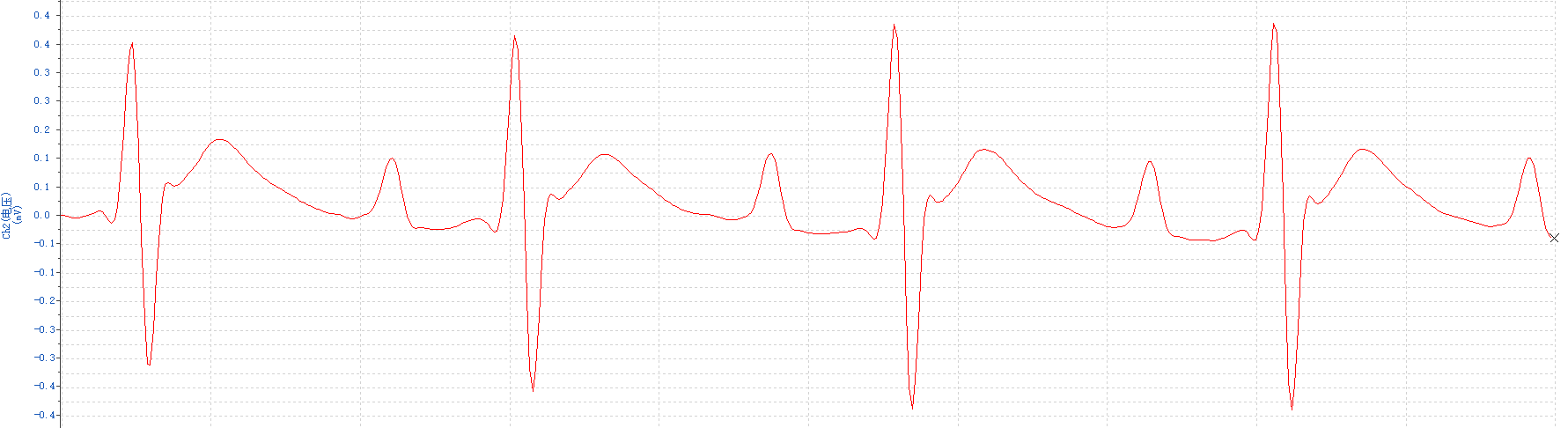


(5) YIN-K group


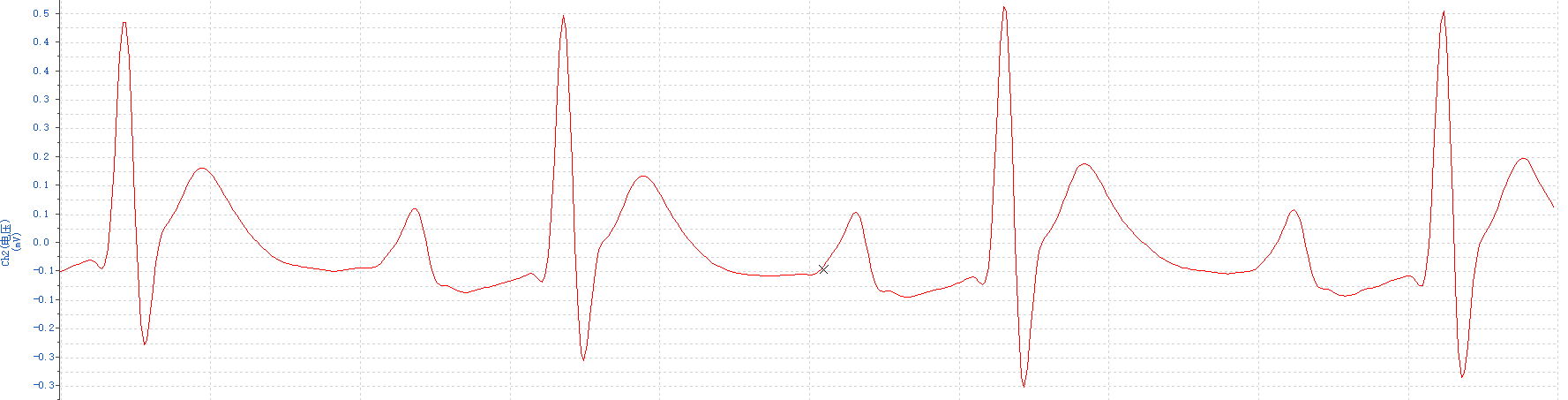


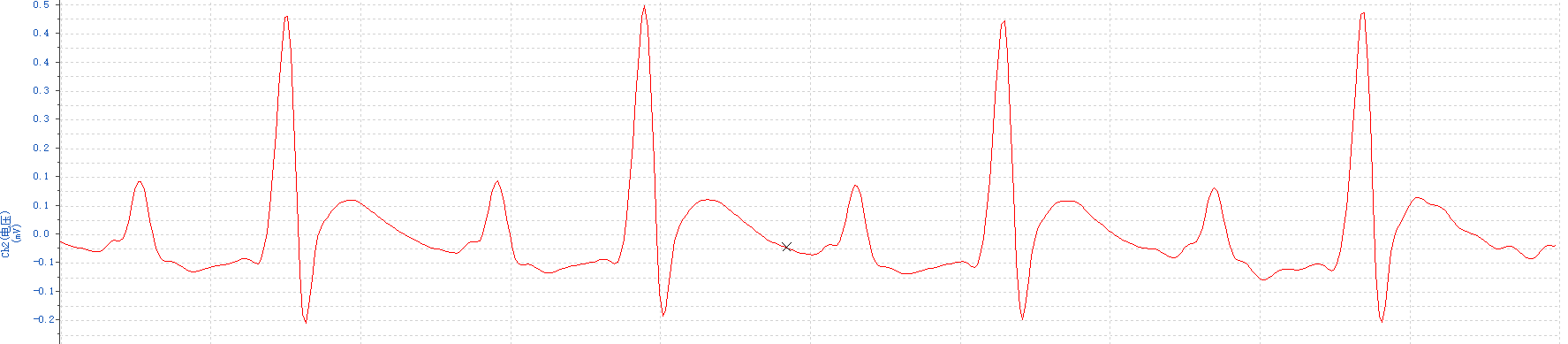


(6) YANG-X group


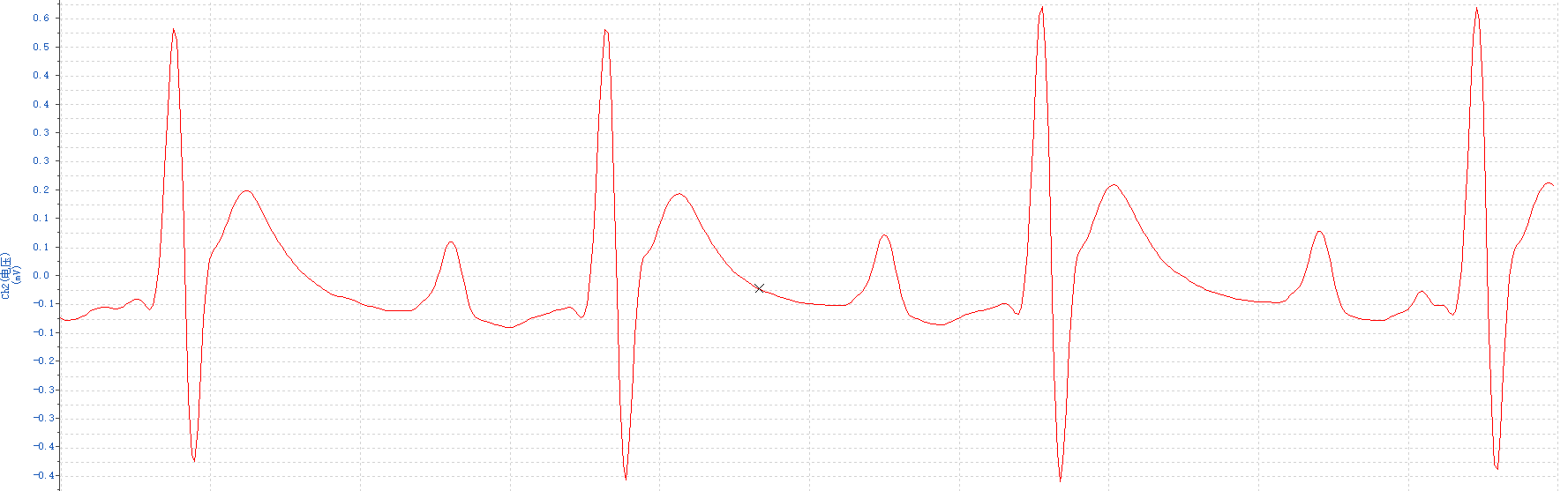


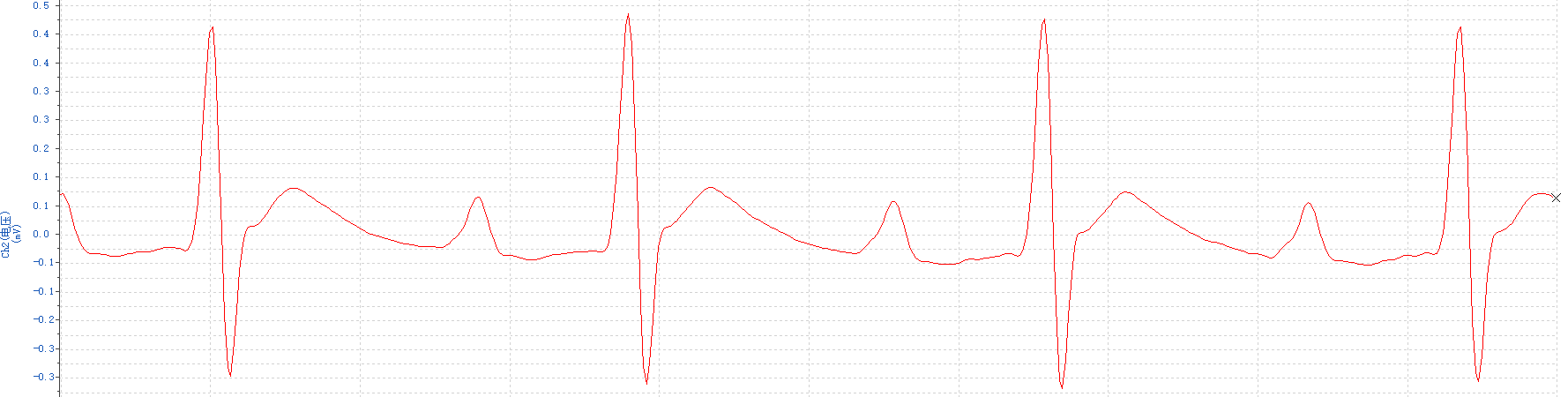


(7) YANG-D group


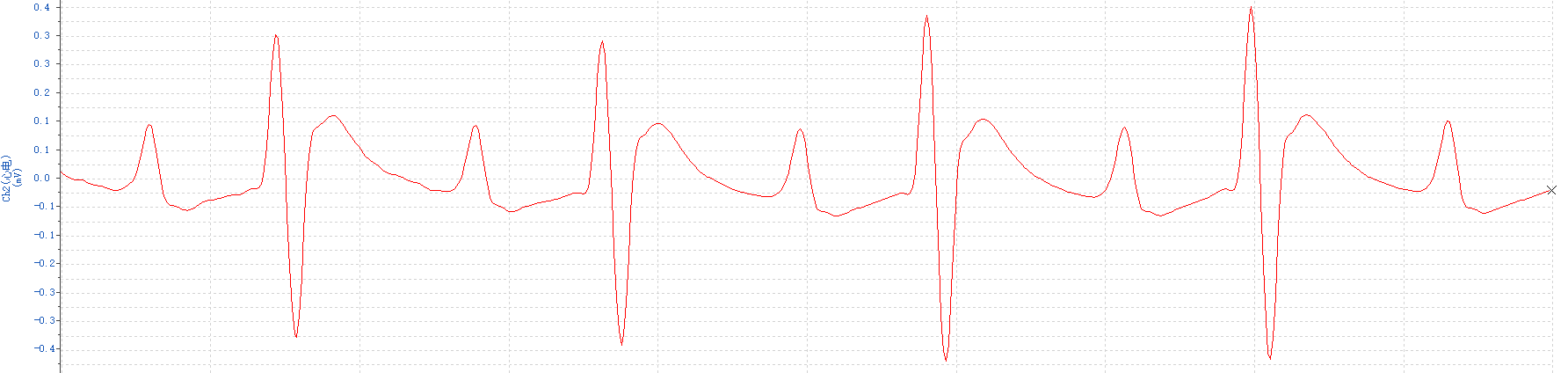


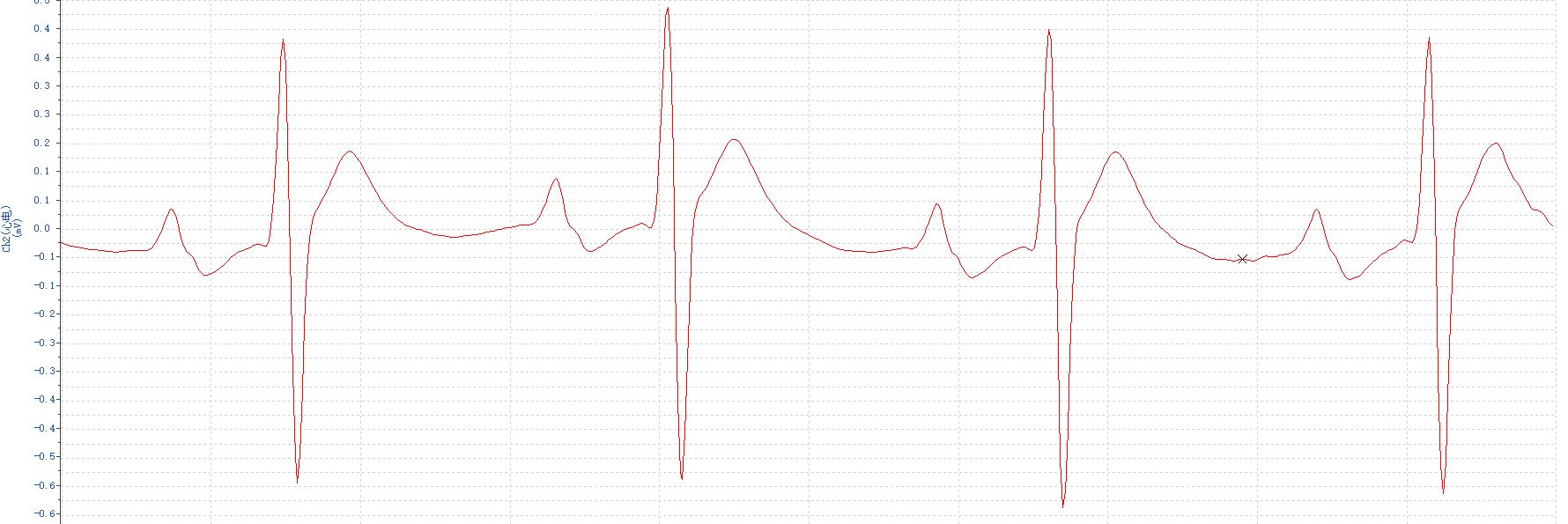


(8) YANG-G group


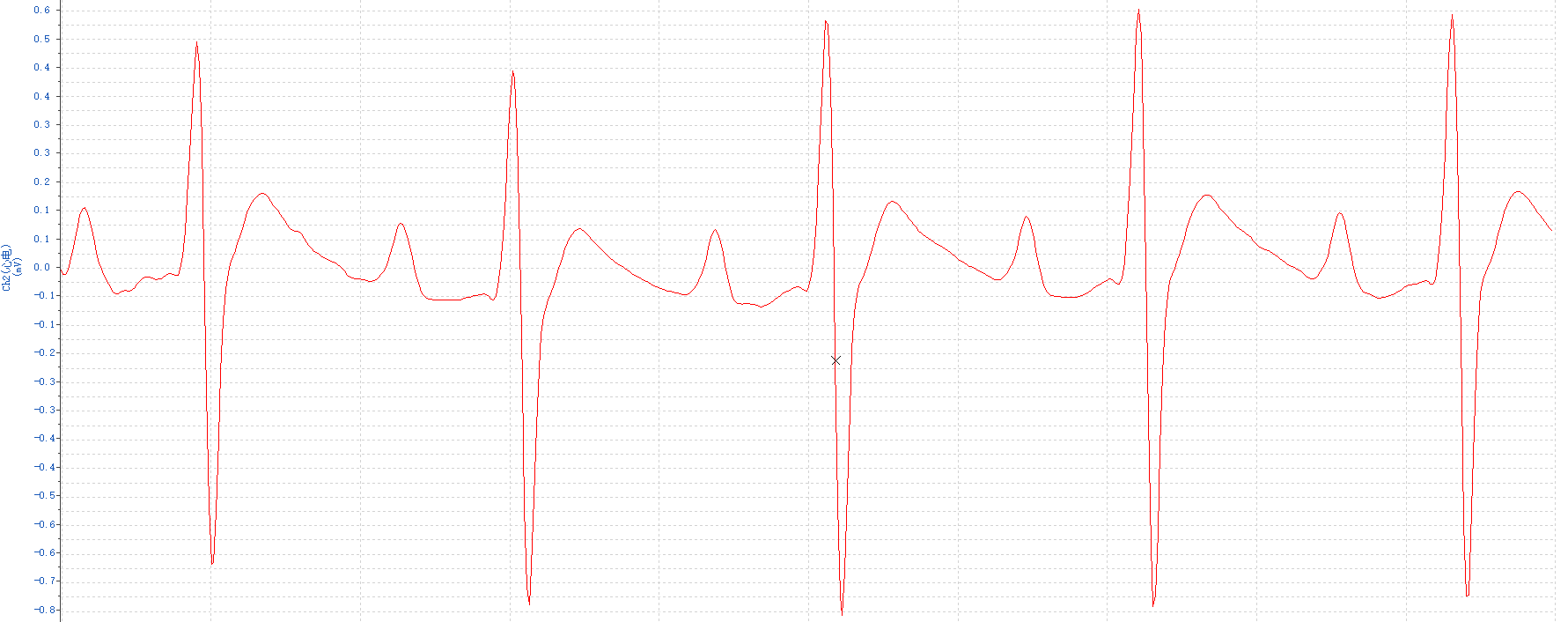


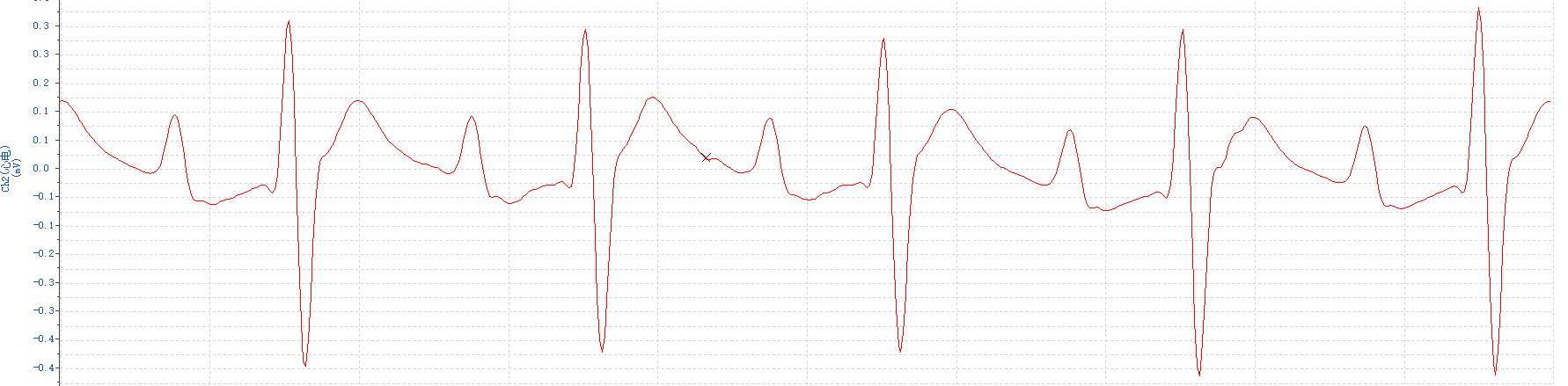


**Figure S3.** The ECG of the rats in different groups.

# Cardiac histology

(1) YANG-K group (left×200, Scale 100 μm, right×400, Scale 50 μm)


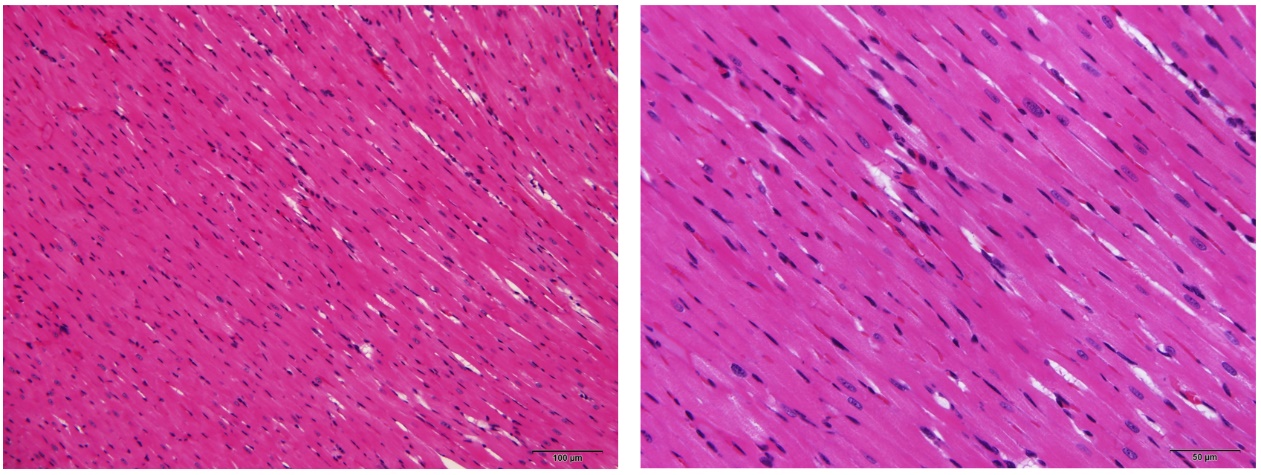


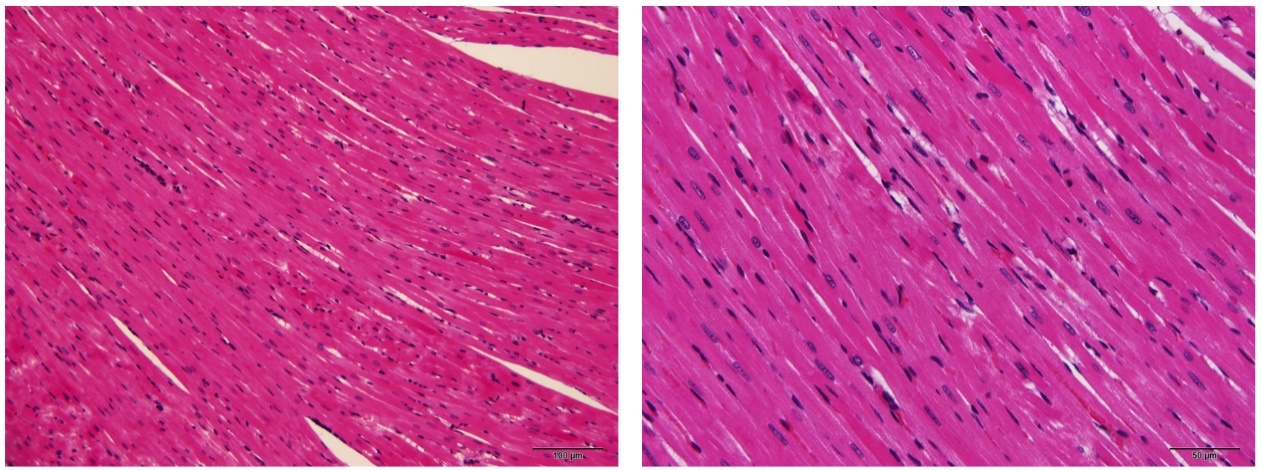


(2) YANG-X group (left×200, Scale 100 μm, right×400, Scale 50 μm)


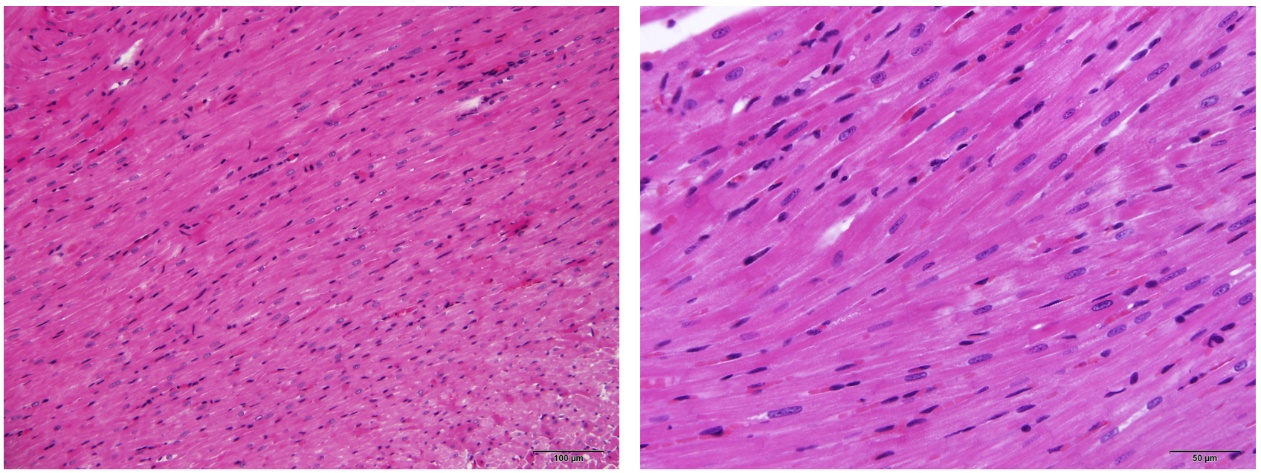


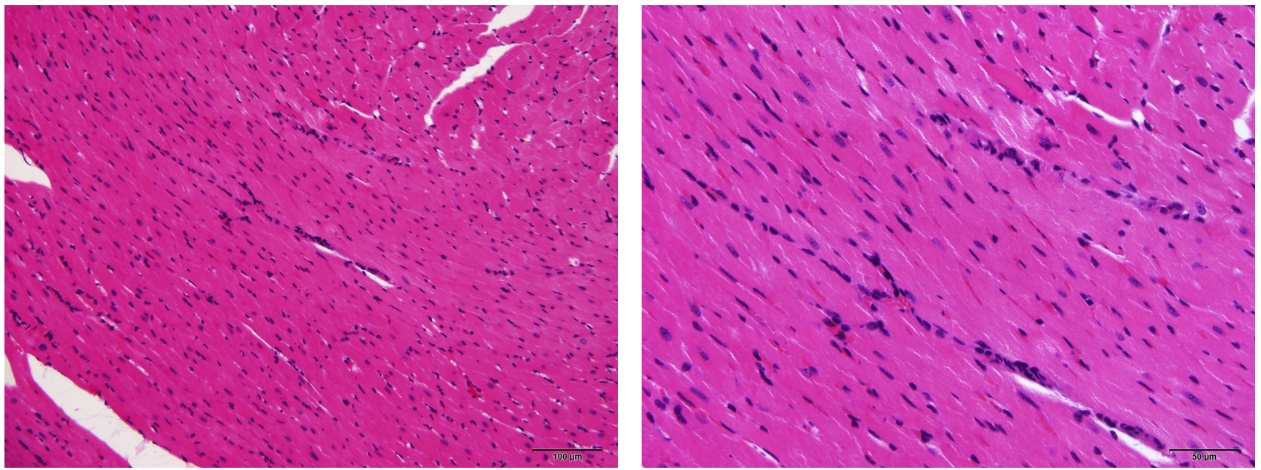


(3) YANG-D group (left×200, Scale 100 μm, right×400, Scale 50 μm)


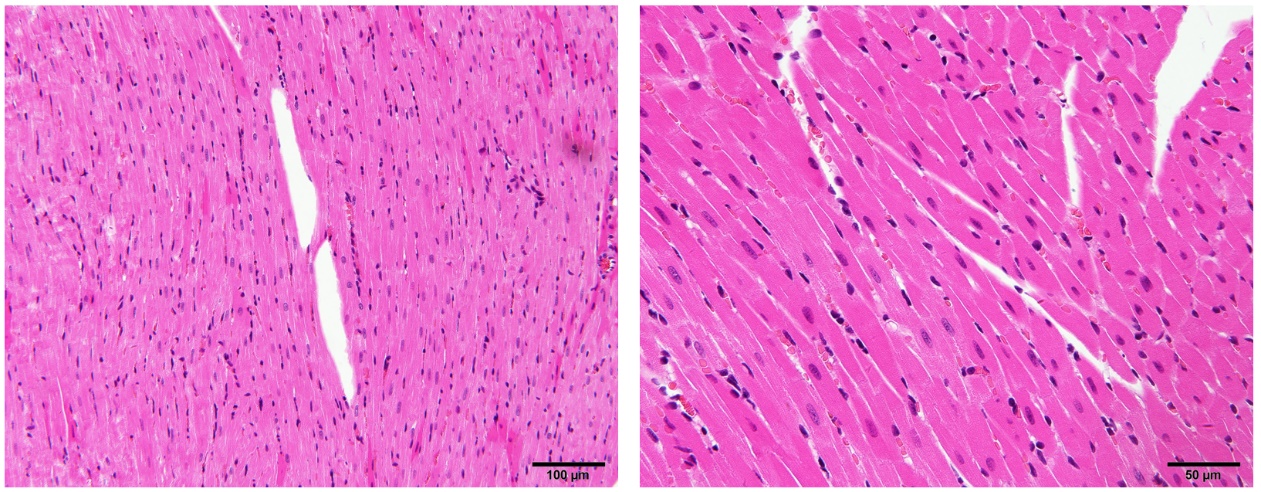


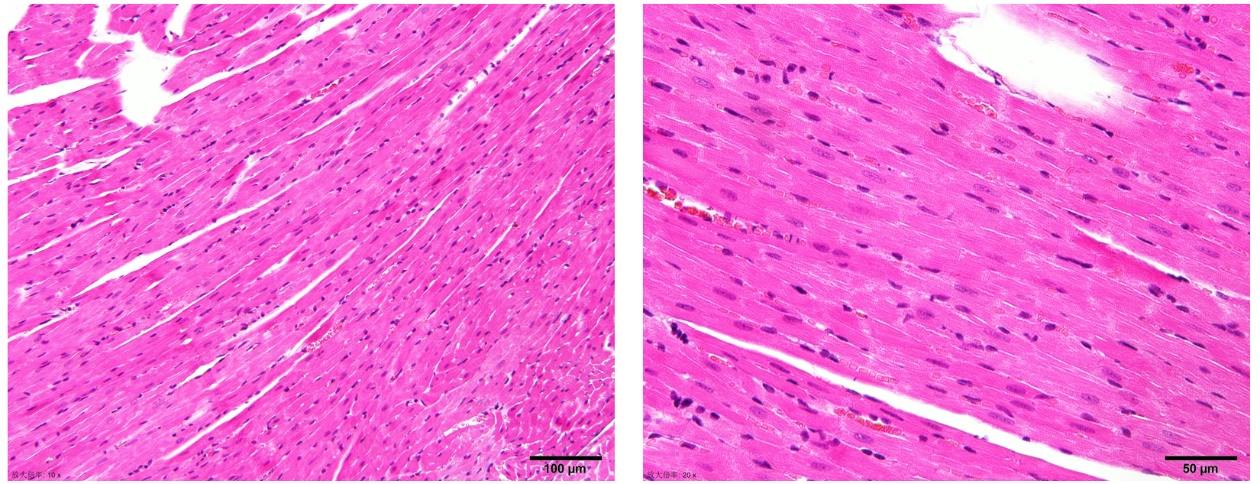


(4) YANG-G group (left×200, Scale 100 μm, right×400, Scale 50 μm)


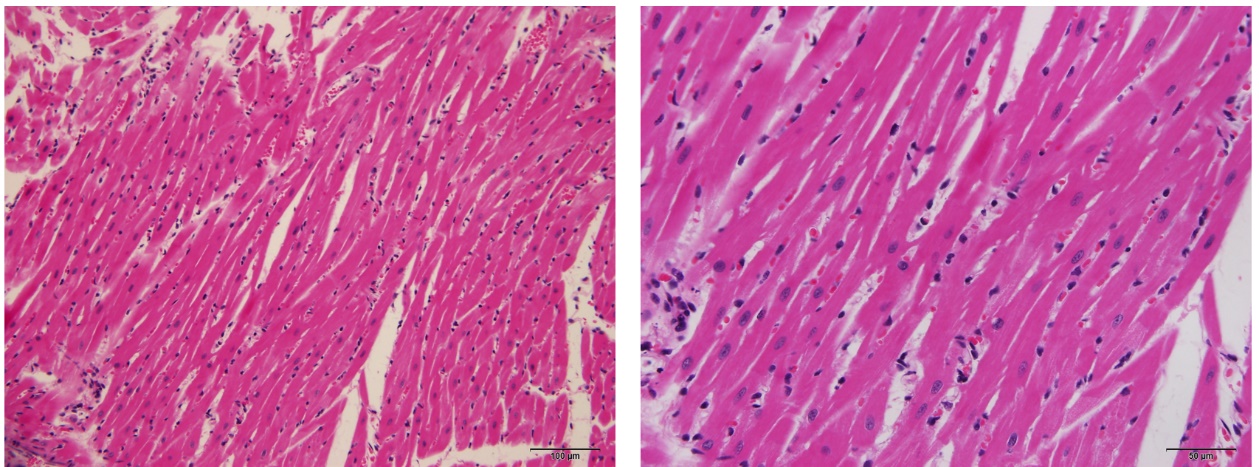


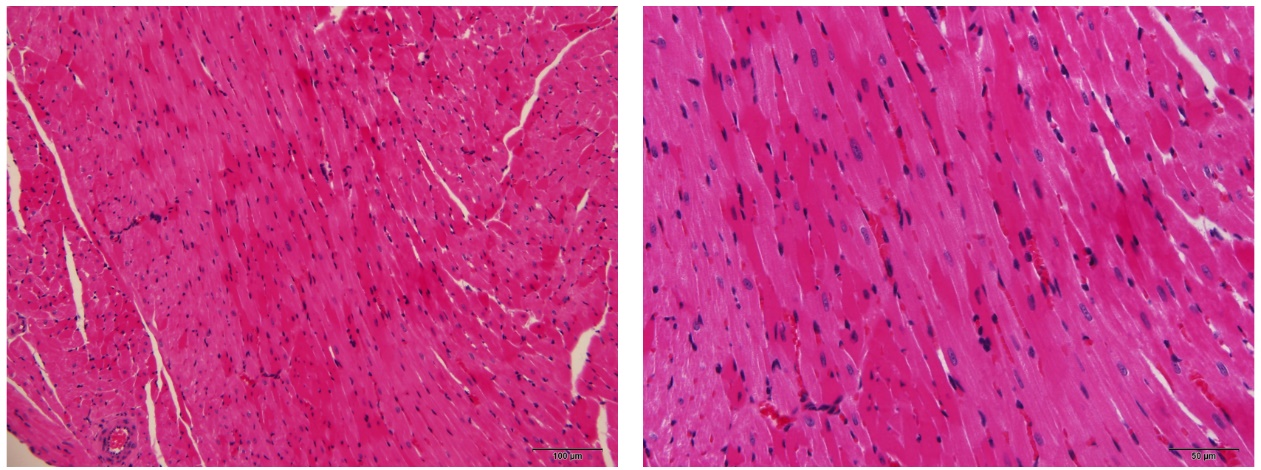


(5) YIN-K group (left×200, Scale 100 μm, right×400, Scale 50 μm)


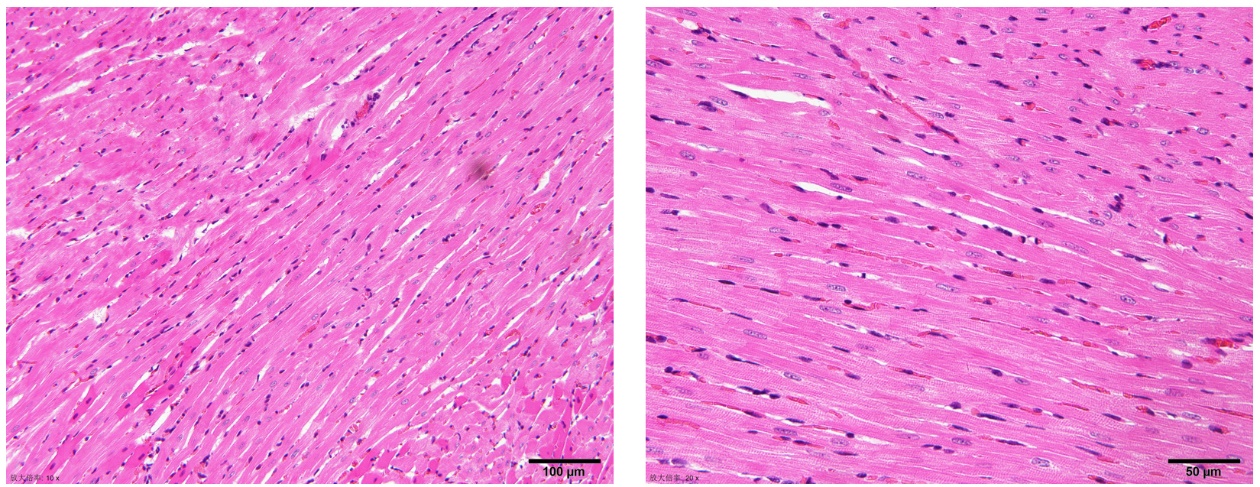


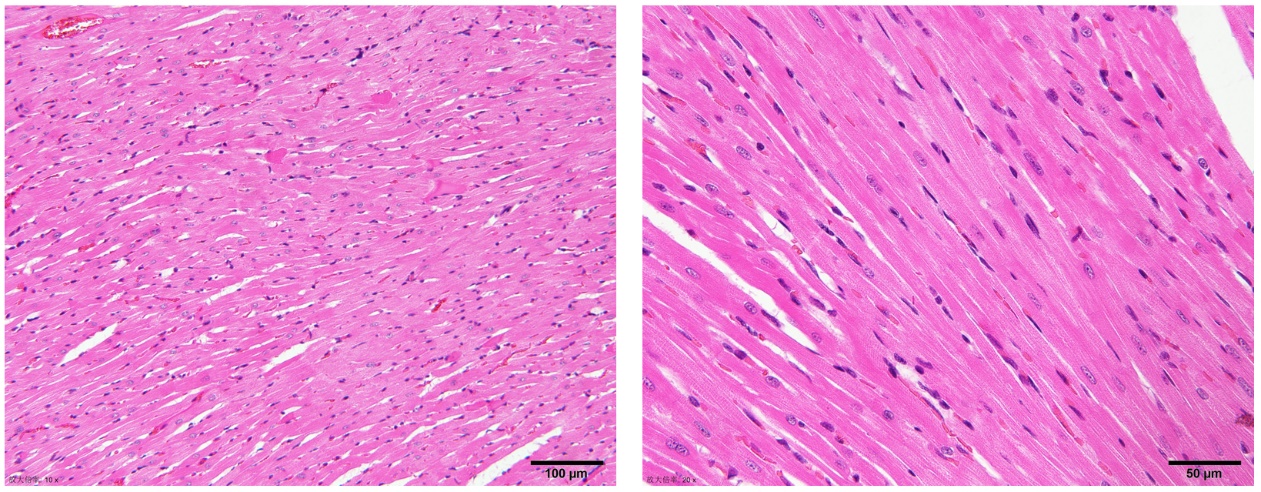


(6) YIN-X group (left×200, Scale 100 μm, right×400, Scale 50 μm)


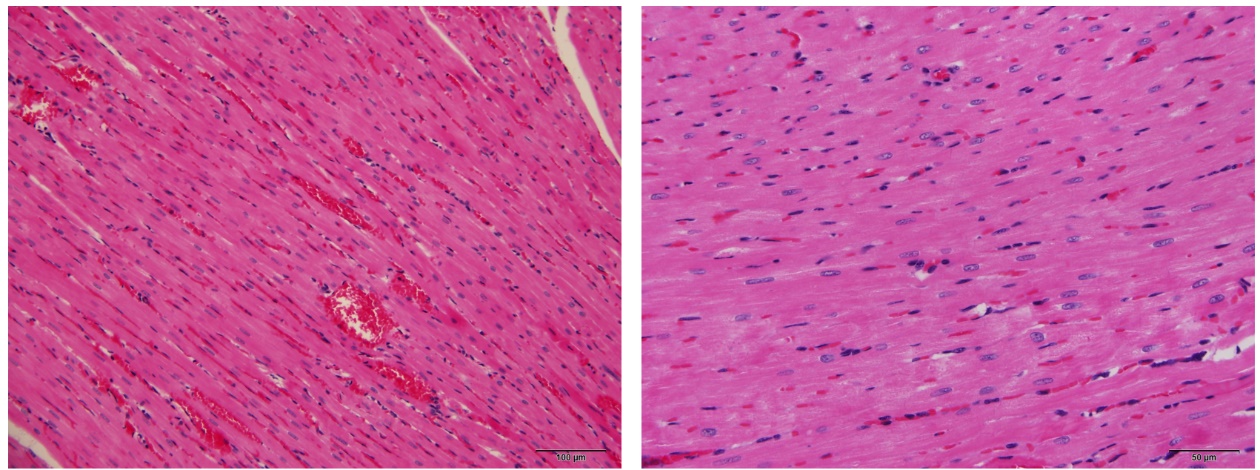


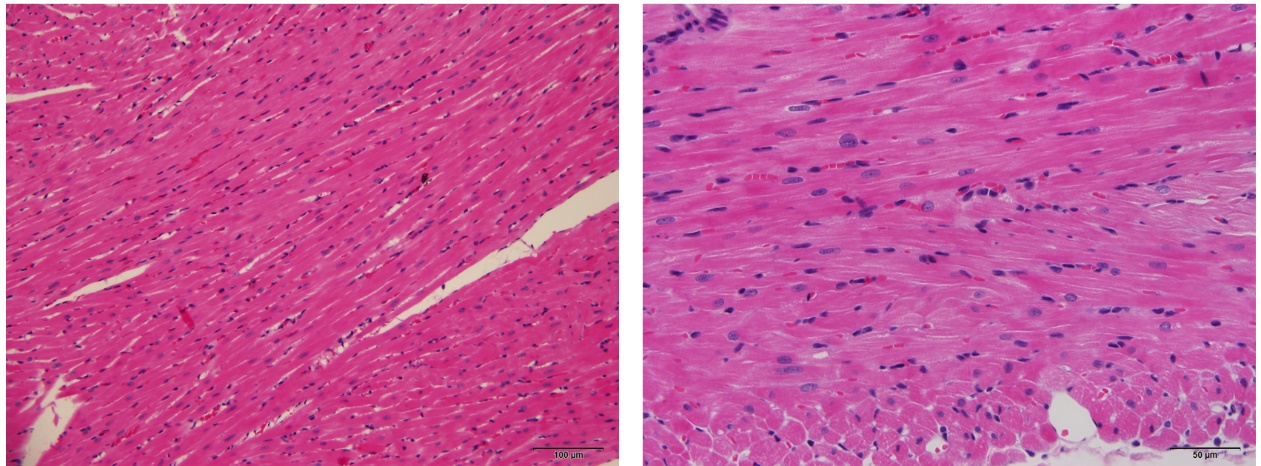


(7) YANG-D group (left×200, Scale 100 μm, right×400, Scale 50 μm)


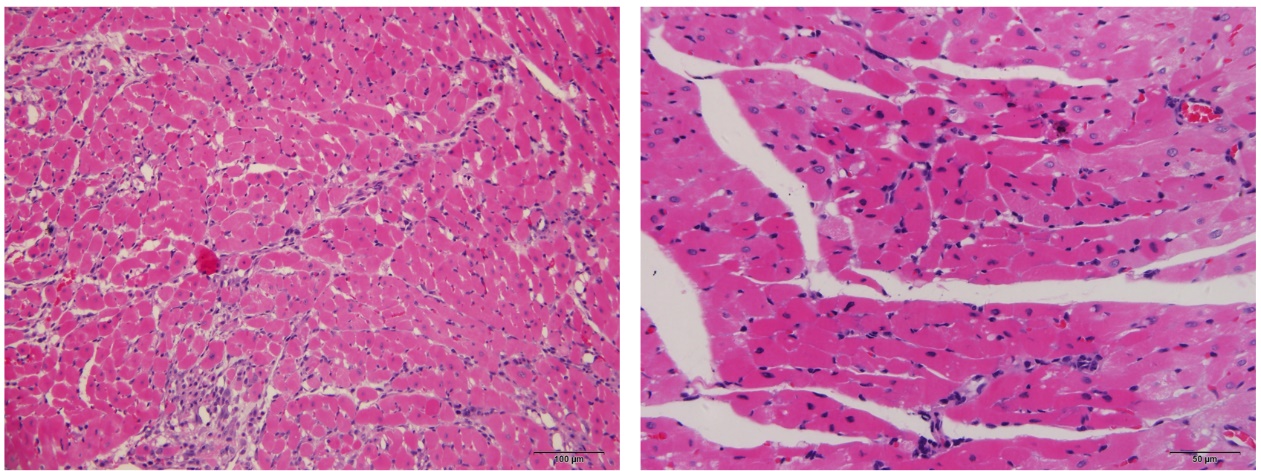


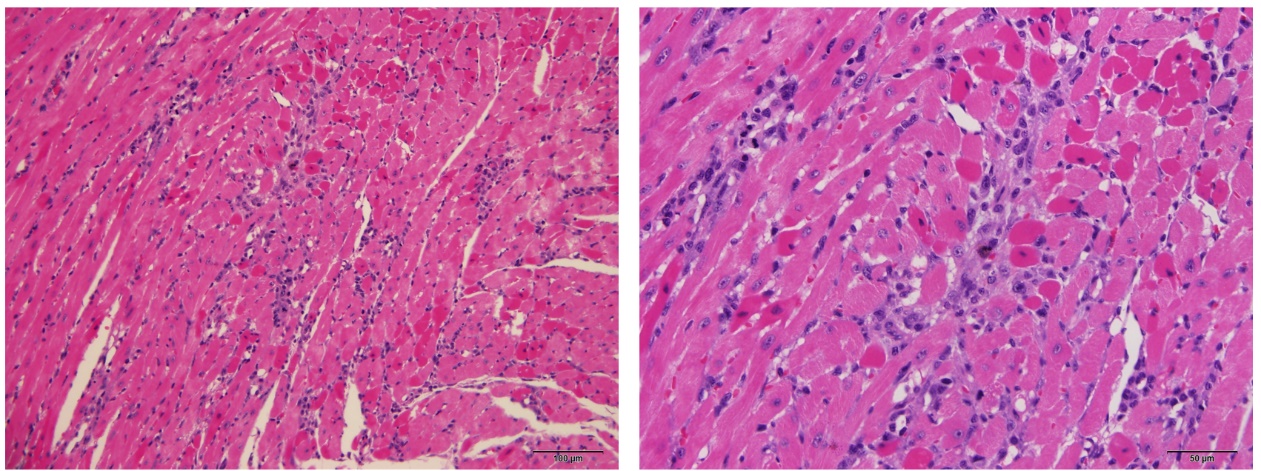


(8) YIN-G group (left×200, Scale 100 μm, right×400, Scale 50 μm)


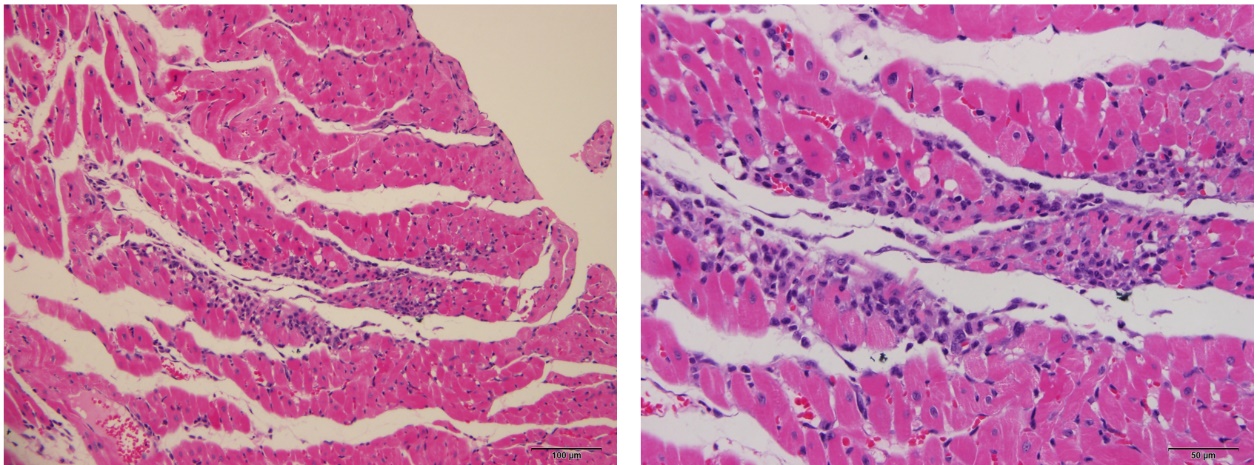


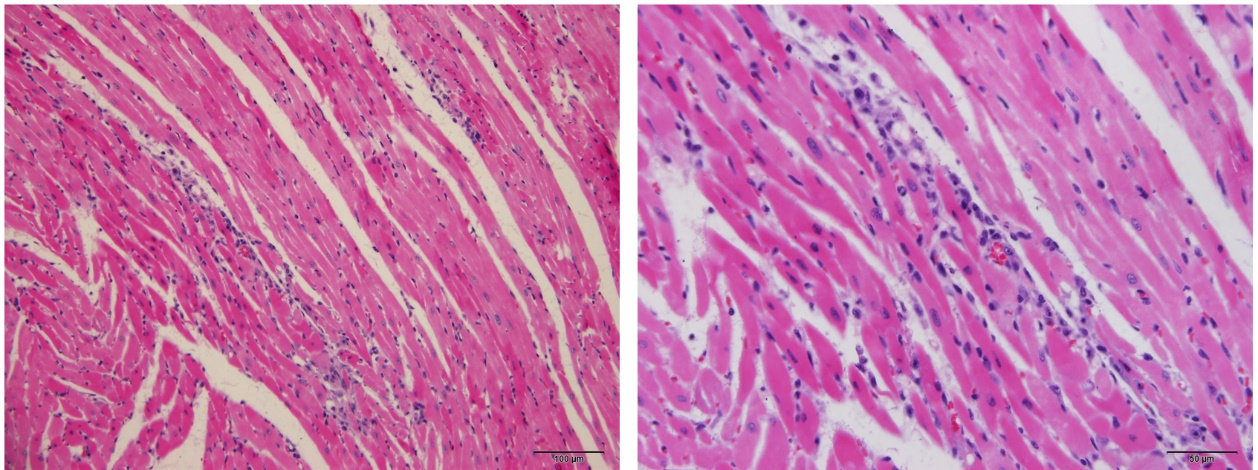


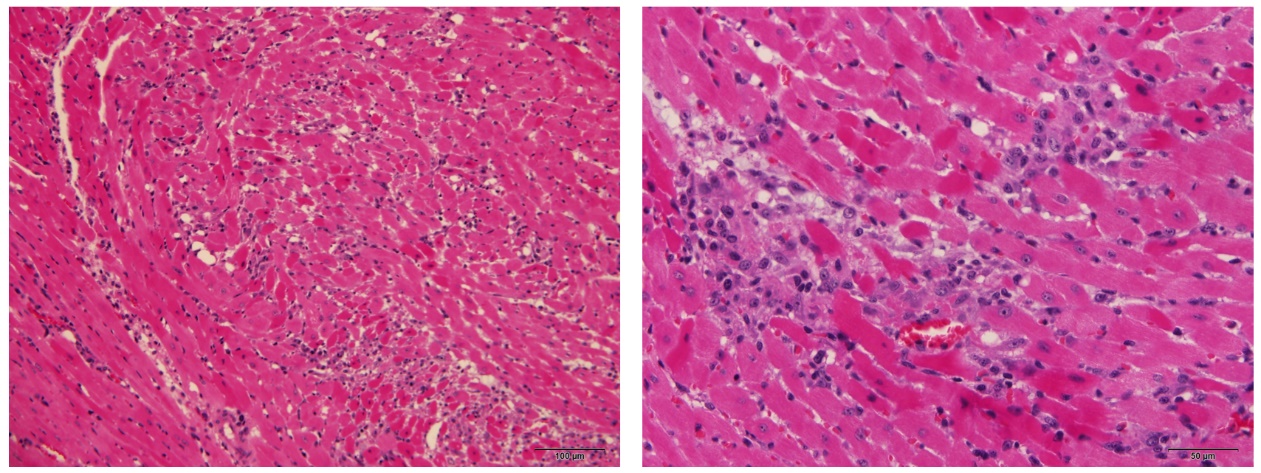


**Figure S4.** Cardiac histology of the rats in different groups.

# The protein expression of PKG in heart issue

**Table S6.** The protein expression of PKG in heart issue (*n* = 3, ‾*x* ± *s*)

| **Group** | **PKG/GAPDH** |
| --- | --- |
| YANG-K | 0.79 ± 0.092 |
| YANG-X | 0.81 ± 0.087 |
| YANG-D | 0.80 ± 0.082 |
| YANG-G | 0.56 ± 0.076*^▲^ |
| YIN-K | 0.76 ± 0.078 |
| YIN-X | 0.78 ± 0.077 |
| YIN-D | 0.49 ± 0.087*^▲^ |
| YIN-G | 0.42 ± 0.066*^▲^ |

Note: Compared with the corresponding control group, **P <* 0.05; compared with the corresponding model group, ^▲^*P <* 0.05.


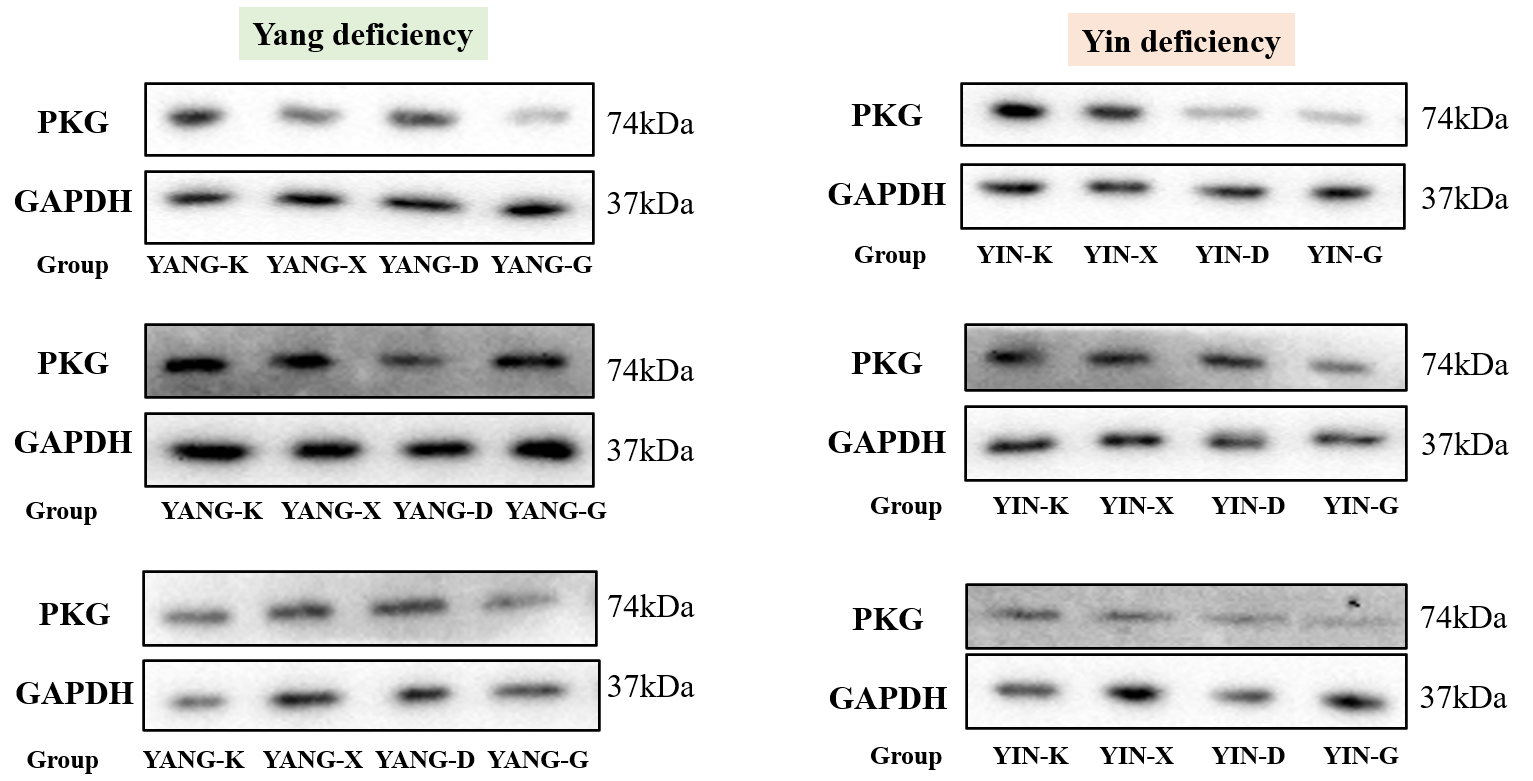


**Figure S5.** The protein expression of PKG in the rats with different groups.

# The results of multivariate data analysis

**Table S7.** The results of multivariate data analysis

|  | **Category** | **Parameter** | **YK *vs* YG** |
| --- | --- | --- | --- |
| ESI^+^ | PCA | R^2^X | 0.457 |
|  |  | Q^2^ | 0.0573 |
|  | OPLS-DA | R^2^X | 0.395 |
|  |  | R^2^Y | 0.931 |
|  |  | Q^2^ | 0.754 |
|  | Permutations | R^2^ | (0.0, 0.857) |
|  |  | Q^2^ | (0.0, -0.411) |
| ESI^-^ | PCA | R^2^X | 0.443 |
|  |  | Q^2^ | -0.00145 |
|  | OPLS-DA | R^2^X | 0.381 |
|  |  | R^2^Y | 0.944 |
|  |  | Q^2^ | 0.765 |
|  | Permutations | R^2^ | (0.0, 0.764) |
|  |  | Q^2^ | (0.0, -0.514) |


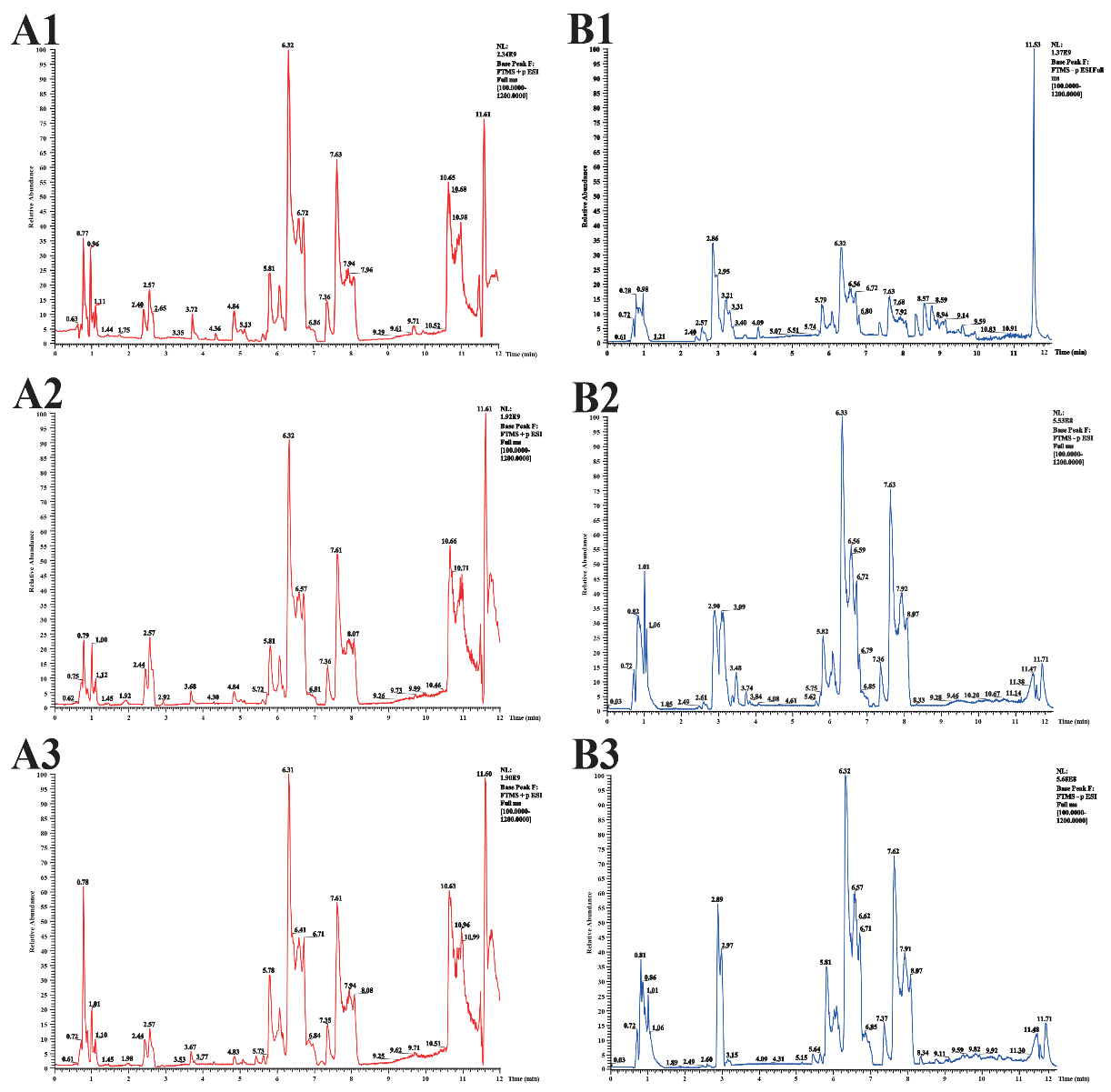


**Figure S6.** Ion flow diagram of serum samples in rats.

Note: A1: QC samples-ESI^+^; A2: YIN-K group-ESI^+^; A3: YIN-G group-ESI^+^; B1: QC samples-ESI^-^; B2: YIN-K group-ESI^-^; B3:YIN-G group-ESI^+^.

# Identification results of differential metabolites

**Table S8.** Identification results of differential metabolites by mass spectrometry

| **No.** | **RT** | **Ion mode** | **Metabolites** | **Obsed *m/z*** | **Caled *m/z*** | **Error *ppm*** | **Formula** | **MS/MS** | **Trends** |
| --- | --- | --- | --- | --- | --- | --- | --- | --- | --- |
| 1 | 0.90 | [M+H]^+^ | Glutaminylglutamic acid | 276.11957 | 276.11901 | 2.03 | C_10_H_17_N_3_O_6_ | 130.05[M+H-C_5_H_8_NO_3_]^+^,84.04[M+H-C_4_H_6_NO]^+^,101.07[M+H-C_4_H_9_N_2_O]^+^ | ↑** |
| 2 | 0.93 | [M+H]^+^ | D-Proline | 116.07093 | 116.07061 | 2.76 | C_5_H_9_NO_2_ | 70.07[M+H-C_4_H_8_N]^+^,116.07[M+H-C_5_H_9_NO_2_]^+^ | ↑** |
| 3 | 0.98 | [M+H]^+^ | Deoxycytidine | 228.09758 | 228.09788 | -1.32 | C_9_H_13_N_3_O_4_ | 112.05[M+H-C_4_H_6_N_3_O]^+^,69.05[M+H-C_3_H_5_N_2_]^+^ | ↓** |
| 4 | 1.00 | [M+K]^+^ | 5-Hydroxyisourate | 185.03049 | 185.03053 | -0.22 | C_5_H_4_N_4_O_4_ | 142.02[M+K-C_4_H_4_N_3_O_2_]^+^,114.03[M+K-C_3_H_4_N_3_O_2_]^+^,71.02[M+K-C_2_H_3_N_2_O]^+^ | ↓* |
| 5 | 1.00 | [M+H]^+^ | Glutamylthreonine | 249.10713 | 249.10811 | -3.94 | C_9_H_16_N_2_O_6_ | 84.04[M+H-C_4_H_6_NO]^+^,74.06[M+H-C_3_H_8_NO]^+^,120.07[M+H-C_4_H_10_NO_3_]^+^,102.0550[M+H-C_4_H_8_NO_2_]^+^ | ↑* |
| 6 | 1.01 | [M+H]^+^ | Cytosine | 112.05083 | 112.05054 | 2.59 | C_4_H_5_N_3_O | 112.05[M+H-C_4_H_5_N_3_O]^+^,95.02[M+H-C_4_H_3_N_2_O]^+^,69.01[M+H-C_2_HN_2_O]^+^ | ↓* |
| 7 | 1.01 | [M+Na]^+^ | Uric acid | 169.03560 | 169.03562 | -0.12 | C_5_H_4_N_4_O_3_ | 169.04[M+Na-C_5_H_4_N_4_O_3_]^+^,126.03[M+Na-C_4_H_4_N_3_O_2_]^+^ | ↓* |
| 8 | 1.02 | [M-H]^-^ | Xanthosine | 283.06873 | 283.06846 | 1.32 | C_10_H_12_N_4_O_6_ | 151.03[M-H-C5H3N4O2]^-^,108.02[M-H-C_4_H_2_N_3_O]^-^,283.07[M-H-C_10_H_12_N_4_O_6_]^-^ | ↓* |
| 9 | 1.10 | [M+NH_4_]^+^ | D-Lysine | 147.11296 | 147.11280 | 1.09 | C_6_H_14_N_2_O_2_ | 72.08[M+NH_4_-C_4_H_10_N]^+^,84.08[M+NH_4_-C_5_H_10_N]^+^ | ↑* |
| 10 | 1.11 | [M+K]^+^ | Xanthine | 153.04079 | 153.04070 | 0.59 | C_5_H_4_N_4_O_2_ | 53.04[M+K-C_2_HN_2_]^+^,135.03[M+K-C_5_H_3_N_4_O]^+^, 110.04[M+K-C_4_H_4_N_3_O]^+^ | ↓* |
| 11 | 1.55 | [M+H]^+^ | Indoleacetaldehyde | 160.07602 | 160.07569 | 2.06 | C_10_H_9_NO | 160.08[M+H-C_10_H_9_NO]^+^,142.07[M+H-C_10_H_8_N]^+^ | ↑* |
| 12 | 2.86 | [M+H]^+^ | Ribose 1,5-bisphosphate | 348.94412 | 348.94296 | 3.24 | C_5_H_12_O_11_P_2_ | 73.03[M+H-C_3_H_5_O_2_]^+^,115.04[M+H-C_5_H_7_O_3_]^+^ | ↑** |

| 13 | 2.90 | [M+Br]^-^ | Trichloroethanol glucuronide | | 322.94962 | | 322.94881 | | 2.52 | | C_8_H_11_C_l3_O_7_ | | 110.94[M+Br-C_2_HCl_2_O]^-^,84.99[M+Br-C_3_HO_3_]^-^  59.01[M+Br-C_2_H_3_O_2_]^-^,71.01[M+Br-C3H3O2]^-^ | | | ↑** | |
| --- | --- | --- | --- | --- | --- | --- | --- | --- | --- | --- | --- | --- | --- | --- | --- | --- | --- |
| 14 | 2.90 | [M-H]^-^ | Citraconic acid | | 175.02382 | | 175.02389 | | -0.40 | | C_5_H_6_O_4_ | | 85.03[M-H-C_4_H_5_O_2_]^-^,71.01[M-H-C_3_H_3_O_2_]^-^ | | | ↑*** | |
| 15 | 3.03 | [M+Cl]^-^ | Indole-5,6-quinone | | 146.02365 | | 146.02379 | | -0.96 | | C_8_H_5_NO_2_ | | 118.03[M+Cl-C_7_H_4_NO]^-^,146.02[M+Cl-C_8_H_4_NO_2_]^-^ | | | ↓* | |
| 16 | 3.13 | [M-H]^-^ | Glutamylcysteine | | 328.98416 | | 328.98462 | | -1.40 | | C_8_H_14_N_2_O_5_S | | 59.01[M-H-C_2_H_3_O_2_]^-^,85.03[M-H-C_4_H_5_O_2_]^-^ | | | ↑*** | |
| 17 | 3.70 | [M+H]^+^ | trans-2-Dodecenoylcarnitine | | 342.26382 | | 342.26389 | | -0.20 | | C_19_H_35_NO_4_ | | 85.03[M+H-C_4_H_5_O_2_]^+^,60.08[M+H-C_3_H_10_N]^+^, 144.10[M+H-C_7_H_14_NO_2_]^+^ | | | ↑* | |
| 18 | 3.71 | [M+H]^+^ | Palmitic amide | | 256.26318 | | 256.26349 | | -1.21 | | C_16_H_33_NO | | 256.26[M+H-C_16_H_33_NO]^+^,86.06[M+H-C_4_H_8_NO]^+^, 100.07[M+H-C_5_H_10_NO]^+^ | | | ↑* | |
| 19 | 3.72 | [M+NH_4_]^+^ | Phytosphingosine | | 318.29962 | | 318.30027 | | -2.04 | | C_18_H_39_NO_3_ | | 318.30[M+NH_4_-C_18_H_37_O_3_]^+^,57.0704[M+NH_4_-C_4_H_9_]^+^,300.2903[M+NH_4_-C_18_H_38_NO_2_]^+^ | | | ↑* | |
| 20 | 3.94 | [M+H]^+^ | 3, 5-Tetradecadiencarnitine | | 368.27939 | | 368.27954 | | -0.41 | | C_21_H_37_NO_4_ | | 85.03[M+H-C_4_H_5_O_2_]^+^,60.08[M+H-C_3_H_10_N]^+^, 144.10[M+H-C_7_H_14_NO_2_]^+^,189.16[M+H-C_14_H_21_]^+^ | | | ↑* | |
| 21 | 4.32 | [M-H]^-^ | 1-Stearoylglycerophosphoserine | | 544.30377 | | 544.30395 | | -0.33 | | C_24_H_48_NO_9_P | | 78.96[M-H-O_3_P]^-^,153.00[M-H-C_3_H_6_O_5_P]^-^ | | | ↓** | |
| 22 | 5.21 | [M-H]^-^ | LysoPE(0:0/20:3) | | 562.31439 | | 562.31470 | | -0.55 | | C_25_H_46_NO_7_P | | 78.96[M-H-O_3_P]^-^,153.00[M-H-C_3_H_6_O_5_P]^-^ | | | ↓* | |
| 23 | 5.23 | [M-H]^-^ | Deoxycholic acid | | 459.27322 | | 459.27362 | | -0.87 | | C_24_H_40_O_4_ | | 345.24[M-H-C_22_H_33_O_3_]^-^,391.28[M-H-C_24_H_40_  O_4_]^-^ | | | ↓** | |
| 24 | 5.78 | [M-H]^-^ | LysoPE(20:5/0:0) | | 544.26801 | | 544.26717 | | 1.54 | | C_25_H_42_NO_7_P | | 153.00[M-H-C_3_H_6_O_5_P]^-^,78.96[M-H-O_3_P]^-^, 140.01[M-H-C_2_H_7_NO_4_P]^-^,196.04[M-H-C_5_H_11_NO_5_P]^-^ | | | ↓** | |
| 25 | 6.04 | [M+FA-H]^-^ | | LysoPE(18:3/0:0) | | 520.26807 | | 520.26745 | | 1.19 | | C_23_H_42_NO_7_P | | 153.00[M+FA-H-C_3_H_6_O_5_P]^-^,78.96[M+FA-H-O_3_P]^-^,96.97[M+FA-H-H_4_O_4_P]^-^ | ↓** | |  |
| 26 | 6.08 | [M+Hac-H]^-^ | | LysoPC(P-18:1/0:0) | | 550.35217 | | 550.35050 | | 3.04 | | C_26_H_52_NO_6_P | | 78.9591[M+Hac-H-O_3_P]^-^,401.2462[M+Hac-H-C_21_H_38_O_5_P]^-^ | ↓* | |  |
| 27 | 6.44 | [M-H]^-^ | | Erinacine A | | 431.24060 | | 431.24146 | | -2.00 | | C_25_H_36_O_6_ | | 89.02[M-H-C_3_H_5_O_3_]^-^,59.01[M-H-C_2_H_3_O_2_]^-^ | ↓** | |  |
| 28 | 6.63 | [M-H]^-^ | | LysoPE(P-16:0/0:0) | | 504.27142 | | 504.27189 | | -0.93 | | C_21_H_44_NO_6_P | | 78.96[M-H-O3P]^-^  436.28[M-H-C_21_H_43_NO_6_P]^-^,140.01[M-H-C_2_H_7_NO_4_P]^-^,196.04[M-H-C_5_H_11_NO_5_P]^-^ | ↓** | |  |
| 29 | 6.69 | [M+H]^+^ | | LysoPC(20:2/0:0) | | 548.37085 | | 548.37107 | | -1.08 | | C_28_H_54_NO_7_P | | 184.07[M+H-C_5_H_15_NO_4_P]^+^,87.00[M+H-C_5_H_12_N]^+^, 60.08[M+H-C_3_H_10_N]^+^ | ↓* | |  |
| 30 | 6.88 | [M+FA-H]^-^ | | LysoPE(20:2/0:0) | | 504.30963 | | 504.30992 | | -0.58 | | C_25_H_48_NO_7_P | | 307.26[M+FA-H-C_20_H_35_O_2_]^-^,78.96[M+FA-H-O_3_P]^-^,140.01[M+FA-H-C_2_H_7_NO_4_P]^-^, 196.04[M+FA-H-C_5_H_11_NO_5_P]^-^ | ↓** | |  |
| 31 | 6.92 | [M-H]^-^ | | LysoPE(22:2/0:0) | | 532.34027 | | 532.34116 | | -1.67 | | C_25_H_48_NO_7_P | | 78.96[M-H-O_3_P]^-^,140.01[M-H-C_2_H_7_NO_4_P]^-^ | ↓* | |  |
| 32 | 7.19 | [M+Hac-H]^-^ | | LysoPC(18:1/0:0) | | 580.36407 | | 580.36542 | | -2.33 | | C_26_H_52_NO_7_P | | 295.26[M+Hac-H-C_19_H_35_O_2_]^-^,78.96[M+Hac-H-O_3_P]^-^,520.34[M+Hac-H-C_26_H_51_NO_7_P]^-^ | ↓* | |  |
| 33 | 7.64 | [M-H]^-^ | | LysoPA(0:0/18:0) | | 437.26541 | | 437.26662 | | -2.77 | | C_21_H_43_O_7_P | | 78.96[M-H-O_3_P]^-^,153.00[M-H-C_3_H_6_O_5_P]^-^, 96.97[M-H-H_2_O_4_P]^-^ | ↓* | |  |
| 34 | 7.97 | [M-H]^-^ | | LysoPC(0:0/18:0) | | 558.33252 | | 558.33106 | | 2.62 | | C_26_H_54_NO_7_P | | 283.26[M-H-C_18_H_35_O_2_]^-^,78.96[M-H-O_3_P]^-^ | ↓* | |  |

Note: Compared with YIN-K group, **P* < 0.05, ***P* < 0.01, ****P* < 0.001.

# The pathway analysis of differential metabolites

**Table S9.** The pathway analysis of differential metabolites

| **Pathway name** | **Match status** | ***P*** | **Impact** | **Metabolites** |
| --- | --- | --- | --- | --- |
| Purine metabolism | 4/66 | 0.0052 | 0.062 | Xanthine, Xanthosine, Urate, 5-Hydroxyisourate |
| Glycerophospholipid metabolism | 2/36 | 0.060 | 0.16 | 1-Acyl-sn-glycero-3-phosphocholine, Phosphatidate |
| Glycerolipid metabolism | 1/16 | 0.17 | 0.012 | Phosphatidate |
| Sphingolipid metabolism | 1/21 | 0.21 | 0.0041 | Phytosphingosine |
| Phosphatidylinositol signaling system | 1/28 | 0.27 | 0.0015 | Phosphatidate |
| Arginine and proline metabolism | 1/38 | 0.35 | 0.00 | D-Proline |
| Pyrimidine metabolism | 1/39 | 0.36 | 0.0051 | Deoxycytidine |
| Tryptophan metabolism | 1/41 | 0.38 | 0.014 | Indole-3-acetaldehyde |
| Tyrosine metabolism | 1/42 | 0.38 | 0.00 | Indole-5,6-quinone |
| Metabolism of xenobiotics by cytochrome P450 | 1/64 | 0.52 | 0.00 | Trichloroethanol glucuronide |
